# Neuronal types in the mouse amygdala and their transcriptional response to fear conditioning

**DOI:** 10.1038/s41593-023-01469-3

**Published:** 2023-10-26

**Authors:** Hannah Hochgerner, Shelly Singh, Muhammad Tibi, Zhige Lin, Niv Skarbianskis, Inbal Admati, Osnat Ophir, Nuphar Reinhardt, Shai Netser, Shlomo Wagner, Amit Zeisel

**Affiliations:** 1 Faculty of Biotechnology and Food Engineering, Technion—Israel Institute of Technology, Haifa, Israel; 2 Sagol Department of Neurobiology, University of Haifa, Haifa, Israel

**Keywords:** Fear conditioning, Molecular neuroscience

## Abstract

The amygdala is a brain region primarily associated with emotional response. The use of genetic markers and single-cell transcriptomics can provide insights into behavior-associated cell state changes. Here we present a detailed cell-type taxonomy of the adult mouse amygdala during fear learning and memory consolidation. We perform single-cell RNA sequencing on naïve and fear-conditioned mice, identify 130 neuronal cell types and validate their spatial distributions. A subset of all neuronal types is transcriptionally responsive to fear learning and memory retrieval. The activated engram cells upregulate activity-response genes and coordinate the expression of genes associated with neurite outgrowth, synaptic signaling, plasticity and development. We identify known and previously undescribed candidate genes responsive to fear learning. Our molecular atlas may be used to generate hypotheses to unveil the neuron types and neural circuits regulating the emotional component of learning and memory.

## Abstract (web summary)

The neuronal subtypes regulating the emotional component of learning and memory are not fully described. Here the authors provide a molecular atlas of the adult mouse amygdala and show the subsets of cells transcriptionally responsive to fear.

## Main

Learning and the formation of long-term memories require enduring physical changes in neurons—the so-called memory trace, or engram^1^. Neuronal ensembles participating in a specific memory (engram cells) strengthen synaptic connectivity over the course of memory consolidation. This requires the induction of gene expression in response to neuronal activation, for example, of proteins necessary to assemble new synaptic complexes. Engram cells are sparse but necessary and sufficient to support a particular memory and can be identified to support different types of memory in different brain regions. For example, engrams of intrinsic, emotionally associated memories have been studied in the amygdala^2,3^. The amygdala consists of multiple anatomical regions, developmentally attributed to the striatal, cortical (superficial) and subcortical (deep) structures^4^. These subregions are defined by their distinct cytoarchitecture, connectivity and functionality, and have increasingly revealed great diversity in cell-type composition^5–8^. Emotional responses associated with fear learning involve basolateral amygdala (BLA), intercalated amygdala (IA) and central amygdala (CEA) clusters^2,9,10^. Learning paradigms reflecting other, nonaversive valences (for example, appetitive behaviors) have since revealed nuanced amygdala roles in emotional sensing. These alternate functions were often achieved through neuronal populations in the same regional circuitries, distinguished only by different molecular identities^5,11,12^. Other amygdala subregions are studied in social, parenting and aggression behaviors and olfactory integration^7,13^.

Single-cell transcriptomics has achieved detailed cell taxonomies of the nervous system^14–17^, including the basolateral, central and medial subregions of the amygdala^6,18–20^, and has also detected transcriptional signatures of neuronal activation^21–23^, social behaviors^24,25^ and learning and memory^26–28^ in the amygdala and elsewhere. Given that the specificity of memories may be encoded by specific molecular cell types^11,19^, linking the molecular description of amygdala cell types to their functional roles on a more systematic scale is of increasing interest. Therefore, we used unbiased single-cell RNA sequencing (scRNA-seq) on whole cells gently dissociated from the full amygdala in 23 naïve and fear-conditioned mice. We applied parallel approaches to map spatial distributions of the resulting taxonomy of neuronal types to the amygdala’s subregions. Finally, we describe which neuronal types participated in fear learning, and the orchestrated transcriptional response across consolidation and memory recall.

## Results

### The amygdala’s cell-type taxonomy reflects regional divides

**Fig. 1:**
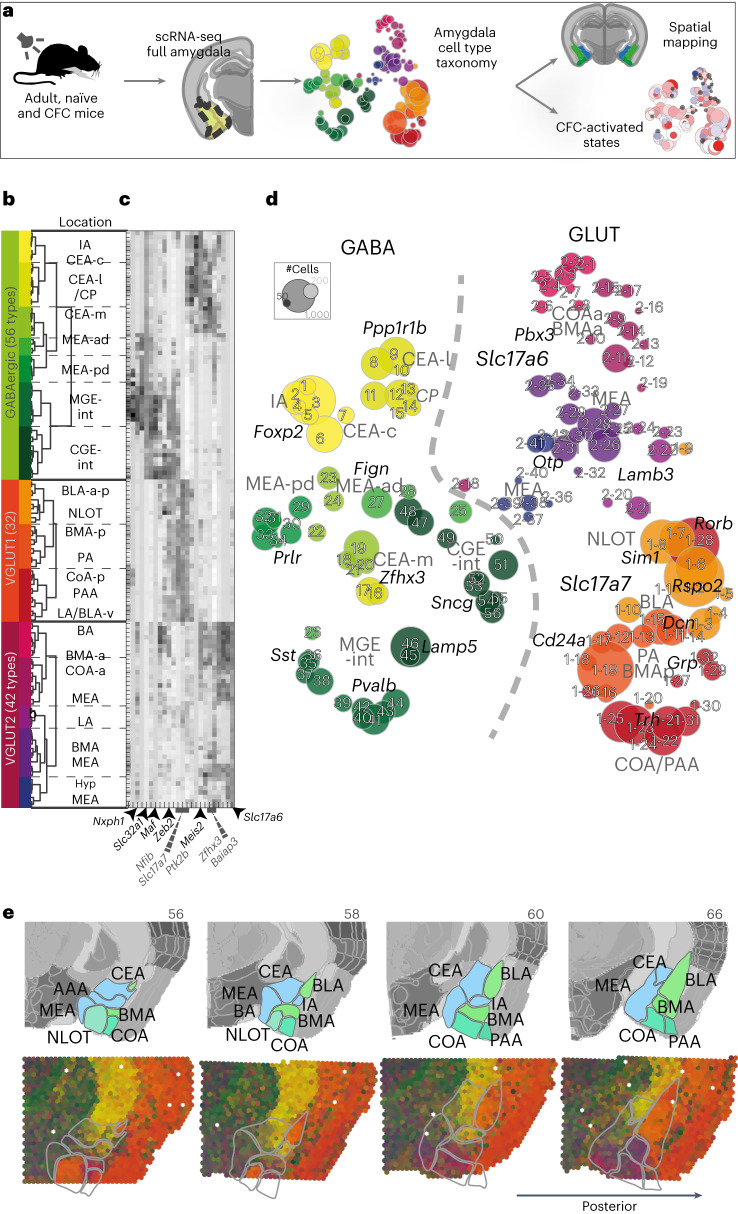
Amygdala neuron taxonomy. **a**, Experiment overview—scRNA-seq of the whole amygdala of 23 mice constructed the cell-type taxonomy that was then spatially mapped to distinct amygdala nuclei and analyzed for CFC response. **b**, Data structure—of 55,514 whole-cell transcriptomes, 30,184 were neurons. Neuronal cell types by dendrogram order per neurotransmitter type (GABAergic (13,006 cells and 56 clusters) or glutamatergic (17,178 cells)), with amygdala location on the right. Glutamatergic cells expressed either vesicle transporter *Slc17a6* (VGLUT2, 5,231 cells and 42 clusters) or *Slc17a7* (VGLUT1, 11,947 cells and 32 clusters), colored by a branch of highly correlated cell types. **c**, Heatmap of genes that were differentially expressed between branches of the dendrogram. **d**, t-SNE visualization of all 130 neuronal types by cluster average, colored by branch; circle area corresponds to cluster size. **e**, Spatial distribution of neuronal types in the amygdala, shown as weighted cell-type correlation with four anterior–posterior coronal sections ST (Visium), colored by branch. Top: corresponding reference sections, adjusted from Allen Reference Atlas—Mouse Brain (atlas.brain-map.org), with amygdala regions and section numbers (top) annotated. Source data

**Extended Data Fig. 1:**
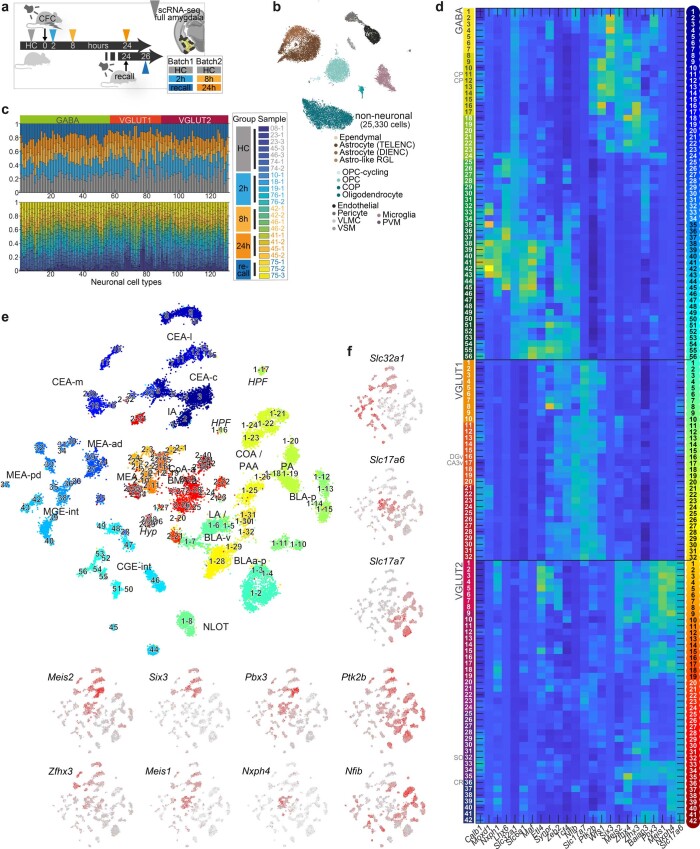
Cell types of the amygdala from adult, behaving mice. **a**, Sampling overview for full-cell scRNA-seq on amygdala of naïve, and cued-fear conditioned mice. **b**, tSNE embedding of 25,330 non-neuronal cells, colored by cell type (14 clusters). **c** Fraction of cells for 130 neuronal types, by sample (top) or experimental group (bottom). **c** Expression of genes differentially expressed between branches of the taxonomy, across 130 neuronal cell types by dendrogram order. Left, branch colors; right, cluster colors. **d**, tSNE embedding of 30,184 neuronal cells, colored by cluster (as **c**), annotated by cluster number, and region. **e**, tSNE embedding of neuronal cells with expression of neuronal class-defining genes *Slc32a1, Slc17a7, Slc17a6*; gray, low; red, high; highlighting GABA, VGLUT1 and VGLUT2 cells respectively. **f**, Expression of genes defining branches of the taxonomy; gray, low; red, high.

We developed a refined dissociation protocol to optimize cell viability for conducting whole-cell scRNA-seq on the amygdala of adult mice, a region we previously found to be particularly sensitive to dissociation stress^14^. As for other cell dissociations from brain tissue, it was important to minimize handling time, and any stress (temperature, enzyme and mechanical) experienced by the cells until scRNA-seq. We used a single buffer formulated for the sodium-sensitive neurons of adult mice (Methods). We verified improved amygdala neuronal viability in suspension, which resulted in the detection of >3,000 UMI and >2,500 genes per neuron. We performed whole-cell scRNA-seq on the full amygdala of 23 adult mice, 16 of which had undergone tone-cued fear conditioning (CFC) (Fig. 1a, Extended Data Fig. 1 and Supplementary Table 1). The dataset revealed 25,330 non-neuronal cells organized into 14 clusters (Extended Data Fig. 1) and great diversity among 30,184 neurons.

Neurons are robustly segregated based on their neurotransmitter identity. The neuronal cell-type taxonomy describes the following three main classes: 56 GABAergic cell types (*Gad1*, *Gad2* and *Slc32a1*, 13,006 cells), 32 VGLUT1 types (*Slc17a7*, 11,947 cells) and 42 VGLUT2 types (*Slc17a6*, 5,232 cells). Each neuron class was organized into branches of related cell types that shared hierarchical and combinatorial expression of (homeodomain) transcription factors (Fig. 1b–d, Extended Data Fig. 1 and Supplementary Table 2).

**Extended Data Fig. 2:**
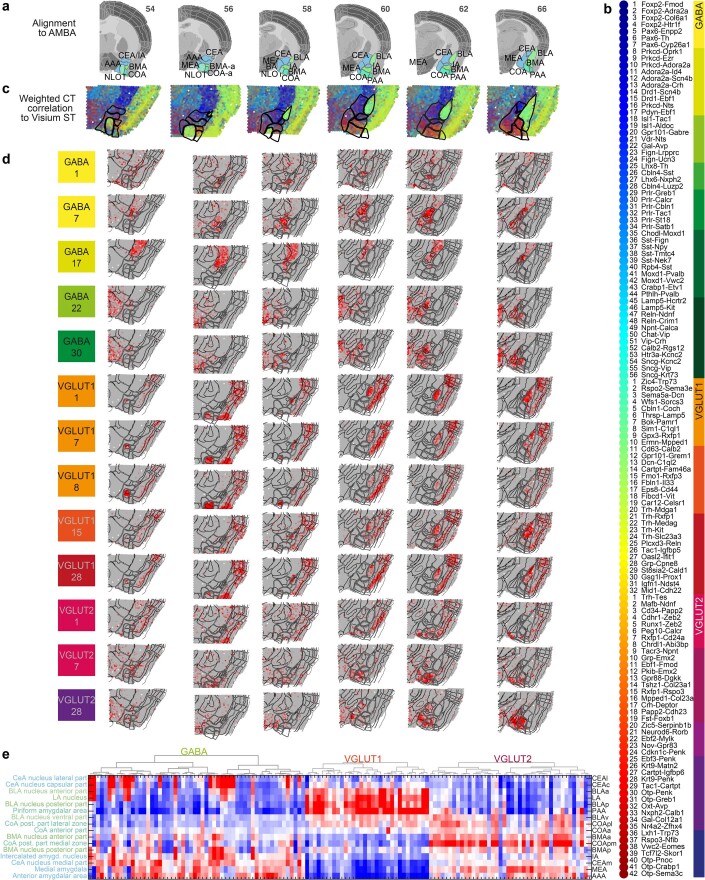
Spatial transcriptomics (Visium) for cell type mapping. **a**, Sample and section overview. 6 coronal sections covering the anterior-posterior amygdala; with alignment to reference sections (adjusted from Allen Reference Atlas – Mouse Brain (atlas.brain-map.org), and amygdala regions highlighted for anatomical reference. **b**, Cell types in dendrogram order, with color map for branches (right), and cluster colors (left). **c**, Spatial mapping of 130 neuronal clusters to Visium ST sections, visualized by weighted correlation of scRNA-seq-derived cell type profiles (**b**) to Visium ST capture spots. **d**, Correlation heatmap between spatial transcriptomics and 13 cell types (examples), visualized on the Visium ST sections. Gray, low; red, high correlation. **e**, Correlation heatmap between spatial transcriptomics expression annotated to amygdala regions (rows, ordered by similarity) and all 130 cell types (dendrogram order, as **b**). Blue, low; red, high correlation. Source data

**Extended Data Fig. 3:**
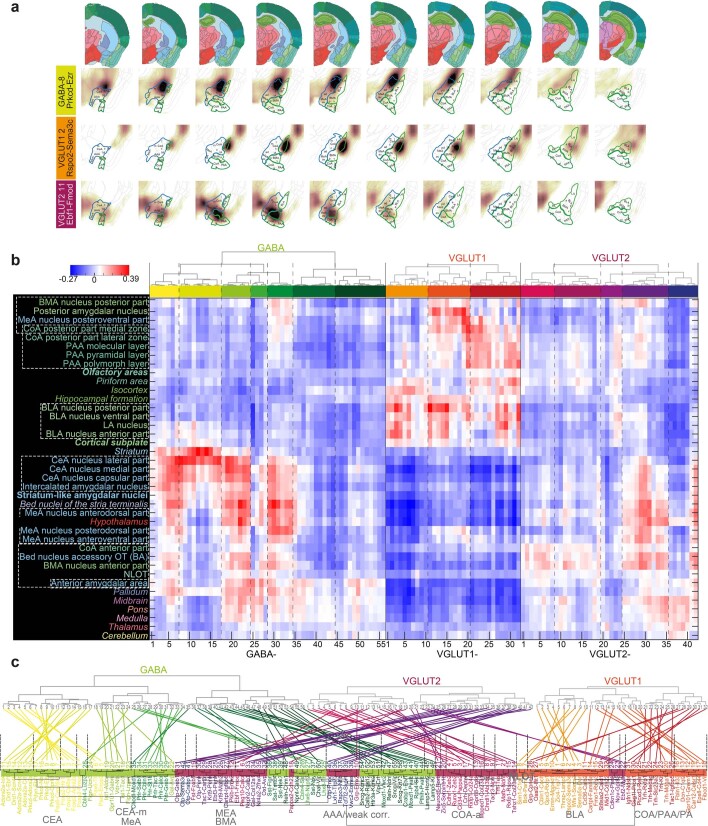
Inferred spatial cell type distributions using AMBA volumetric ISH. **a**, Inferred spatial distribution of three neuronal cell types, by correlating transcriptomic profiles with Allen Mouse Brain Atlas (AMBA-ISH), on eight a-p (left-to-right) sections, with amygdala nuclei labeled. For every cluster and every voxel, a correlation coefficient heatmap is shown (dark, high; white, low). **b**, Spatial correlation of all amygdala neuron clusters with the dissected amygdala nuclei, related or neighboring regions, and other higher-order gray matter structures. Regions are sorted by correlation similarity, cell types by dendrogram order. Region names are colored according to the AMBA convention; regions that were not (fully) dissected are in italic; higher-order amygdala regions are in bold. **c**, Up: Neuronal cell type taxonomy in dendrogram order of scRNA-seq clusters (note; VGLUT2 switched with VGLUT1 compared to Fig. 1), and below; neuronal cell type taxonomy by regional correlation; that is, cell types are arranged according to similarity in correlation with regions analyzed in **b**. The dendrogram is colored by cell class, ‘spatial clusters’ (that is cell types most strongly correlated in their spatial enrichment) with highest correlating regions indicated on dendrogram branches. Cell types are linked between the two classifications to reveal the relation of their scRNA-seq and spatial clustering.

**Extended Data Fig. 4:**
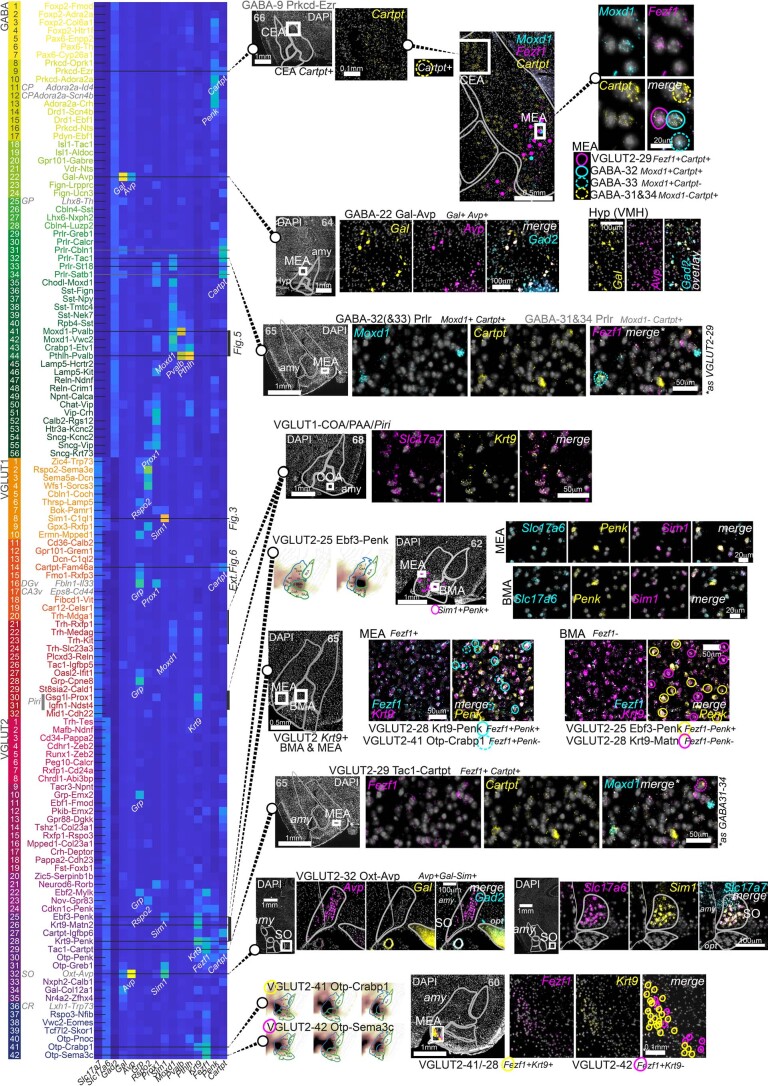
Validation of amygdala cell types using multiplex fluorescent *in situ* hybridization. Left, expression of genes validated *in situ* across the amygdala neuronal cell types taxonomy, in dendrogram order. Cell types validated using combinatorial staining are indicated as lines across the heatmap. Counterstained section overviews indicate position of zoom-ins. Section number in gray indicates anterior-posterior position used for amygdala masks (Allen Reference Atlas – Mouse Brain).

The amygdala is a collection of anatomically and functionally defined nuclei belonging to the following three main structural–developmental regions: striatal, cortical and subplate amygdala nuclei. To reconstruct the molecular cell types’ spatial context, we performed spatial transcriptomics (ST; Visium, 10x Genomics) on coronal sections along the anterior–posterior axis of the amygdala (Fig. 1e and Extended Data Fig. 2). We inferred spatial distributions for each cell type by correlating scRNA-seq expression profiles with this dataset, as well as the Allen Mouse Brain Atlas (AMBA) single-gene in situ hybridization-based volumetric maps^14^ (Extended Data Figs. 2 and 3 and Supplementary Table 3). We manually curated the location of cell types with ambiguous assignment (for example, using multiplex fluorescence in situ hybridization (FISH); Extended Data Fig. 4), and indicated populations sampled from regions neighboring the amygdala. As expected, neuronal classes followed regional borders—VGLUT1 types were enriched in the basolateral and cortical nuclei, whereas VGLUT2 were detected in both cortical and striatal nuclei, with the exception of CEA, which had only GABA types.

Spatial correlation often followed the molecular taxonomy^29^, that is, cell types related by gene expression also shared regional origin, with some interesting exceptions (Extended Data Fig. 3). For example, VGLUT1 clusters that correlated with BLA/lateral amygdala (LA) mixed with a handful of VGLUT2 clusters that localized to the LA. In the medial amygdala (MEA) and basomedial amygdala (BMA), neurons from VGLUT2 and GABA classes intermixed. Spatial correlation analysis also revealed the relations of amygdala cell types to expression profiles found elsewhere in the brain; CEA and IA clusters were molecularly related to their dorsal neighbor caudoputamen (CP), but MEA nuclei were more similar to the bed nuclei of stria terminalis, and hypothalamus—known to share functions and circuitries in social behaviors. Glutamatergic neurons of the subplate and cortical amygdala areas resembled expression profiles also found in the isocortex and hippocampal formation. Inhibitory interneurons lacked spatial enrichment.

### Inhibitory cells mirror projection type and subregion

**Fig. 2:**
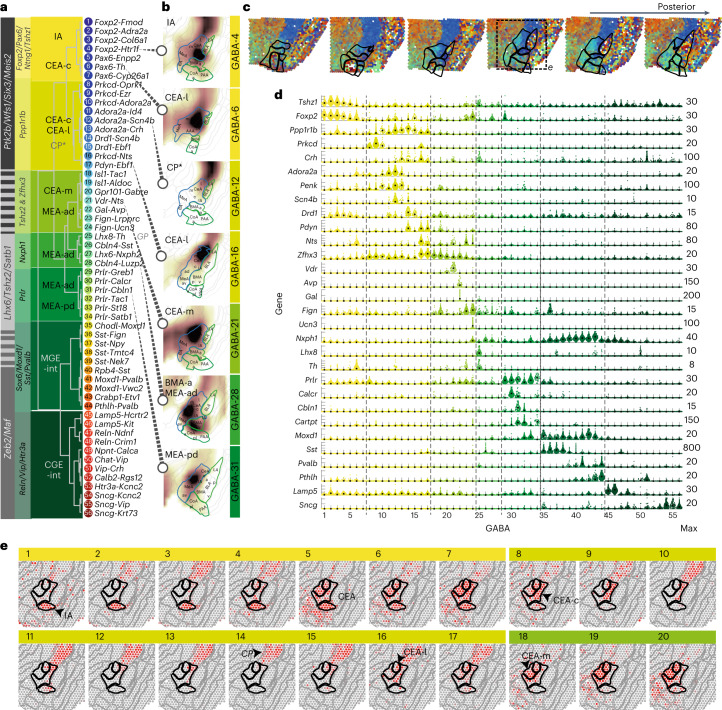
GABAergic cell types of the mouse amygdala. **a**, Dendrogram of GABAergic cell types, with cell-type number and two-gene identifier. Differentially expressed genes defining branching points across the dendrogram (gray), and the specific branches (yellow–green) are highlighted on the left. Likely location, indicated on top of dendrogram (based on AMBA spatial correlation). **b**, Examples of inferred spatial distributions of seven distinct cell types, visualized in a single relevant section with high correlation to AMBA. **c**, Weighted spatial correlation of GABA types to six coronal sections Visium ST. Each dot represents one capture spot, colored by the dominant cell type (see **a**, right). **d**, Genes enriched to single or groups of clusters, visualized as single-cell violin plots. Each dot represents one cell; black square represents the median cluster expression and the number of molecules (*y* scale maximum) is on the right. **e**, GABA cell types 1–20 distributions visualized as correlation heatmap to Visium ST (indicated in **c**). Gray, low and red, high. c, capsular; l, lateral; m, medial; ad, anterodorsal; pd, posterodorsal; int, interneurons.

Amygdala inhibitory cell types are divided into two main branches by their differential expression of *Wfs1*, *Meis2* and *Ptk2b* versus *Maf* and *Zeb2*, enriched on either distal end of the dendrogram (Figs. 1c and 2a and Extended Data Fig. 1). This organization indicated an expected split between projecting neurons of the striatal compartments versus local interneuron populations. Projecting markers *Ptk2b* (telencephalon^14^) and *Zfhx3*, *Zfhx4* and *Meis2* (spinal cord^30^) overlapped with striatal medium spiny neuron (MSN)-like cell types of the IA and CEA. Local/interneuron markers *Nfib*, *Nfix*, *Tcf4*, *Satb1* and *Prox1* (ref. ^30^) were expressed among canonical medial ganglionic eminences (MGE)- and caudal ganglionic eminences (CGE)-derived interneuron types (for example, *Sst*, *Pvalb*, *Vip* or *Sncg*) of the *Maf*/*Zeb2* branch (Fig. 2a–c).

A third branch of cell types in the center of the dendrogram (GABA-22 to GABA-34) expressed *Tshz2*, *Satb1* and *Lhx6*, which overlapped with both distal marker sets. This group was consistent with *Lhx*^+^ MEA-projecting inhibitory cell types^31^ and was rich in neuropeptide expression. For example, GABA-22 coexpressed neuropeptides galanin and vasopressin (*Gal* and *Avp*)^32^ and was located to the MEA-ad (Extended Data Fig. 4). Two types expressed *Fign* and *Nts*, together with either *Lrpprc* and *Th* (GABA-23) or *Tac1* and the peptide hormone precursor *Ucn3*, implied in social behavior^33^ (GABA-24). GABA-26 to GABA-28 expressed GABAergic synapse organizer *Cbln4* and (predicted) neuropeptides *Sst*, *Nxph2* or *Luzp2*. Six distinct GABA populations (GABA-29 to GABA-34) spanned the MEA’s anterior–posterior axis. They expressed receptors for prolactin (*Prlr*), gonadal steroids androgen and estrogen (*Ar* and *Esr2*) and estrogen-responsive *Greb1* and are therefore consistent with populations and pheromone-processing pathways studied in maternal behavior^34–37^. They were molecularly heterogeneous, coexpressing calcitonin receptor *Calcr* (anterior), estrogen receptor *Esr2*, *Dkk3*/*Tac1*/*Cartpt*, *Pappa, St18* or *Moxd1* (posterior; Fig. 2c).

### Inhibitory neurons of valence-learning modulation and output

**Extended Data Fig. 5:**
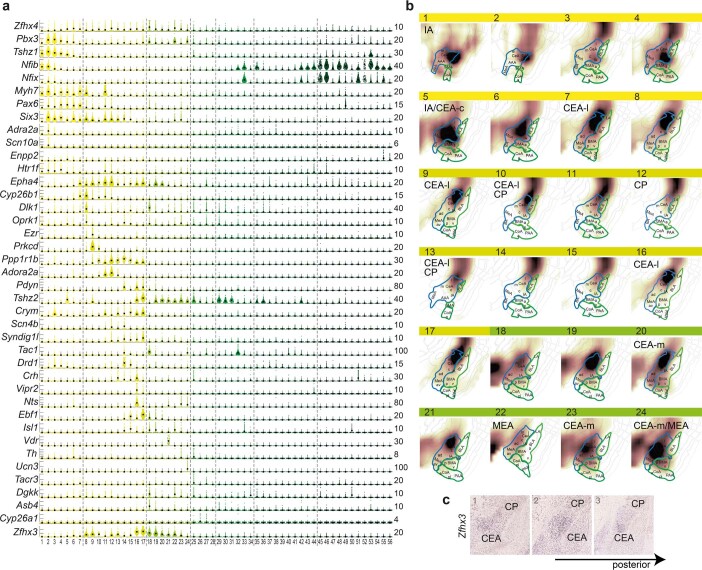
GABAergic populations of intercalated (IA) and central (CEA) amygdala. **a**, Gene expression in IA and CEA populations (GABA1-24), visualized as single-cell violin plots. Each dot represents one cell; black square, median expression, number of molecules (y-scale maximum) on the right. **b**, Inferred spatial distributions of GABA populations 1-24 using AMBA volumetric expression ISH data. Correlation coefficients visualized as a heat map on relevant coronal sections; white, low; brown, high correlation. **c** Expression of *Zfhx3* (Allen Mouse Brain Atlas https://mouse.brain-map.org/experiment/show/74641308) is enriched in the CEA, compared to surrounding and related structures, such as dorsally neighboring striatal caudoputamen (CP).

The IA and CEA have been characterized in detail for their involvement in fear conditioning, and more generally, aversive and appetitive behaviors. They spanned GABA-1 to GABA-21 of the taxonomy and expressed known MSN markers (for example, *Penk*, *Pax6*, *Gpr88* and *Ppp1r1b*) (refs. ^14,38–41^) (Fig. 2a–c, Extended Data Fig. 5 and Supplementary Table 2). Several types followed the D1/D2 convention based on their dopamine receptor expression; however, this was neither an exclusive nor a mutually exclusive hallmark of these neurons. For example, subgroups of a *Ppp1r1b*^+^ CEA population expressed D1-type MSN marker *Drd1* (dopamine receptor 1, GABA-14 and GABA-15), D2 marker *Adora2a* (GABA-10 and GABA-13) or both (GABA-11 and GABA-12; Fig. 2c).

IA types GABA-1 to GABA-4 were embedded between the BLA–CEA regions and expressed *Foxp2*/*Tshz1. Foxp2*-intercalated cells (ITCs)^40^ receive input from the BLA and modulate CEA activity^42^. *Tshz1* was described in a subgroup of D1-MSNs in the nucleus accumbens^39^ and patch-specific MSNs we found in the dorsal and ventral striatum^14^. The *Foxp2* types all expressed *Drd1*, *Myh7* and the serotonin receptor *Htr1f*. ITCs were molecularly distinct based on several genes, which are as follows: *Fmod*, sodium voltage-gated channel *Scn10a* and adrenoreceptor *Adra2a* or *Col6a1*. Notably, like canonical GABAergic interneurons, IA types expressed local-projection-associated genes *Nfib*, *Nfix* and *Tcf4*. GABA-5 to GABA-7 were related to ITCs and expressed the tachykinin receptor *Tacr3*, specifically *Tshz2* and *Enpp2*, *Nts* and *Th* or *Cyp26a1*, but were located in the CEA.

The CEA is a major output region of the amygdala. Most CEA cells (GABA-5 to GABA-21) expressed *Six3*, and *Ano3*, axon guidance-encoding gene *Epha4*, and zinc finger homeobox TF-encoding *Zfhx4* and *Zfhx3*. CEA types GABA-8 to GABA-17 expressed *Ppp1r1b*, encoding the dopamine-dependent central regulatory protein DARPP-32 and *Crym*, described as a marker for MSNs in the medial and caudal striatum^40,43^. The *Ppp1r1b* types correlated with the lateral CEA and neighboring CP (GABA-12, GABA-14 and GABA-15), but *Zfhx3*expression largely distinguished CEA (Fig. 2g and Extended Data Fig. 5). The *Ppp1r1b* branch included well-described CEA neurons positive for protein kinase C δ *Prkcd* (GABA-8 to GABA-10), in combination with kappa-type opioid receptor *Oprk1* (as well as *Dlk1* and *Cyp26b1*), *Ezr* or *Adora2a*. These neurons likely constitute one or several types described for their involvement in both fear and appetitive behaviors^12,44,45^. For example, the smallest of the *Prkcd* cell types, GABA-10, coexpressed *Adora2a* and the calcitonin receptor-like *Calcrl*, which were previously shown in a D2-MSN-like neuron type of the capsular CEA (CEA-c)^12^. Corticotropin-releasing hormone *Crh* (also known as *CRF*) was expressed in D1 and D2 *Ppp1r1b* CEA types GABA-12 to GABA-17, likely marking a separate CEA population studied in anxiogenic circuits, fear response type and memory^46–48^. Several *Crh* types also expressed prodynorphin *Pdyn* and *Isl1*, for instance with *Scn4b* (GABA-14), or *Ebf1, Unc13c* and *Syndig1l* (GABA-17). In contrast with previous reports^44,49^, one D1-type (GABA-16) coexpressed *Prkcd* and *Crh*, as well as *Vipr2* and *Nts*. GABA-17 expressed somatostatin *Sst* and may constitute the CEA-l type reported to antagonize *Crh*-CEA-l neurons in flight-versus-freeze fear behavior^46^. Finally, medial CEA populations (GABA-18 to GABA-21) distinctly expressed the orphan G-protein-coupled receptor *Gpr101*, described in striatal matrix-MSNs^50^. CEA-m subtypes further expressed *Pdyn* and *Isl1* or *Tac1*/*Dlk1*/*Dgkk*/*Asb4,* and GABA-21 highly specifically expressed vitamin D receptor. In sum, CEA populations displayed great molecular diversity. This was in line with recent CEA taxonomies that correlated this diversity with distinct underlying axonal projection patterns^20^ or differential valance encoding^19^.

### Two classes of glutamatergic neurons parcellate the amygdala

**Fig. 3:**
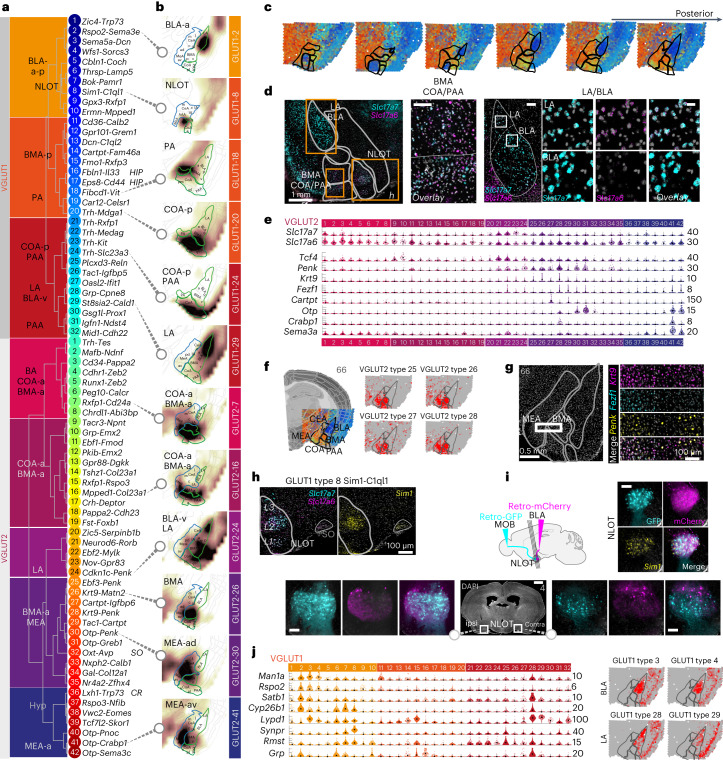
Glutamatergic diversity. **a**, Dendrogram of glutamatergic cell types of the VGLUT1 and VGLUT2 classes, with cluster number and two-gene identifier; likely location indicated on the left. **b**, Examples of inferred spatial distributions of 12 distinct cell types in a relevant coronal section with high correlation to AMBA. Correlation coefficients visualized as a heatmap (white, low and brown, high correlation). **c**, Weighted spatial correlation of GABA types to six coronal sections Visium ST. Each dot represents one capture spot, colored by the dominant cell type (see **a**, right). **d**, Multiplexed fluorescent in situ hybridization of *Slc17a7* (VGLUT1) and *Slc17a6* (VGLUT2) highlighted in LA, BLA (scale bar, 20 μm) and anterior COA/BMA (scale bar, 100 μm) in a single representative anterior section. Top panel section overview: scalebars, 500 μm; LA/BLA overview: scale bar, 100 μm. **e**, VGLUT2 MEA/BMA gene expression visualized as single-cell violin plots. Each dot represents one cell; black square represents median cluster expression and the number of molecules (*y* scale maximum) is on the right. **f**, Examples of related BMA/MEA populations visualized as correlation heatmap to Visium ST (gray, low and red, high). **g**, Multiplex fluorescence in situ hybridization for *Krt9*^+^ MEA/BMA populations, some *Penk*^+^ and coexpressing *Fezf1* in the MEA only. **h**, Multiplex FISH validation of *Slc17a7*^+^*Slc17a6*^+^*Sim1*^+^ cells in the NLOT (overview section in **c**); Scale bar, 100 μm. **i**, NLOT-projection tracing using retro-AAVs to the MOB (hSyn1-EGFP) and BLA (hSyn1-mCherry) reveals NLOT cell labeling ipsi- and contralateral to the injection sites, of *Sim1*^+^ cells (multiplex FISH). Scale bars, overview, 1 mm; zoom-ins, 100 mm. **j**, Left, BLA/LA cell types gene expression; right, examples of spatial distributions. Gene expression visualized as single-cell violin plots, each dot represents one cell; black square represents median expression and number of molecules (*y*-scale maximum) are on the right. BLA/LA populations distribution visualized as correlation heatmap to Visium ST (gray, low and red, high). SO, supraoptic nucleus.

Excitatory amygdala neurons split into two main branches, VGLUT1 and VGLUT2, which were marked by expression of *Slc17a7*, *Slc30a3* and *Tcf4*, or *Slc17a6*, *Slc6a1* and *Baiap3*, respectively (Fig. 1c). VGLUT2 neurons were the more molecularly diverse class; from 5,231 VGLUT2 cells, we identified 42 distinct cell types, while 11,947 VGLUT1 cells yielded 32 distinct cell types (Fig. 3a). Cell types belonging to either group showed spatial enrichment along the anterior–posterior axis and to amygdala subregions; VGLUT1 posterior in BMA, BLA/LA, piriform amygdalar area (PAA), posterior amygdala (PA) and posterior cortical amygdala (COA-p) and VGLUT2 anterior in BMA, anterior cortical amygdala (COA-a), bed nucleus of the accessory olfactory tract (BA) and MEA (Fig. 3a–c). *Slc17a6*^*+*^*Slc17a7*^*+*^ double-positive types were restricted to distinct regions, such as the LA, COA and the nucleus of the lateral olfactory tract (NLOT; Fig. 3d,h).

**Extended Data Fig. 6:**
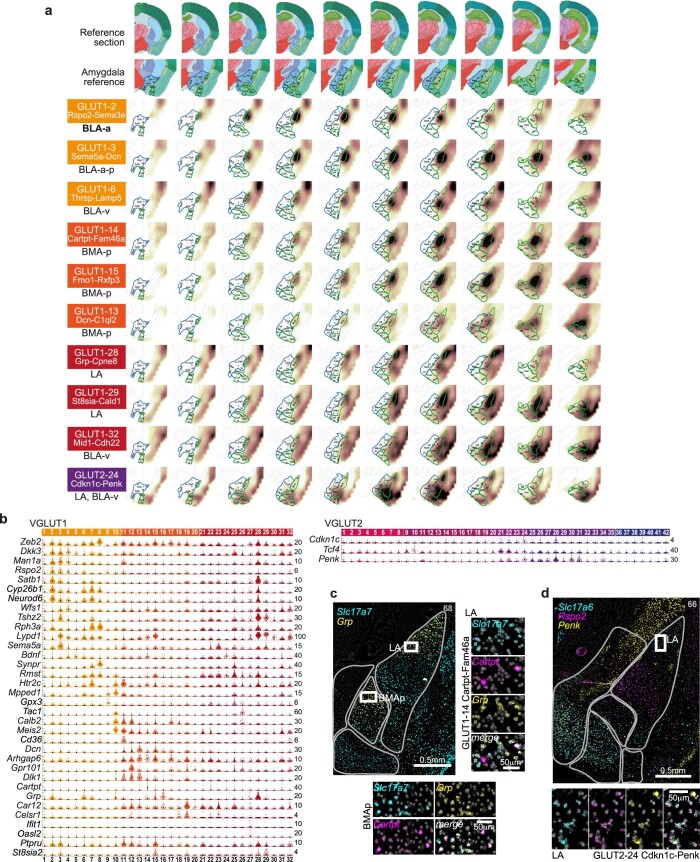
BLA/BMA/LA glutamatergic neuron populations. **a**, Inferred spatial distributions of VGLUT1 populations using AMBA volumetric expression ISH data. Correlation coefficients visualized as a heat map on coronal sections; white, low; brown, high correlation. Top rows, reference section adapted from Allen Reference Atlas – Mouse Brain, and annotated zoom-in; left column, cluster name, and most enriched structure. **b**, BLA/BMA/LA cell types gene expression visualized as single-cell violin plots. Each dot represents one cell; black square, median cluster expression, number of molecules (y-scale maximum) on the right. **c**, Multiplex fluorescent *in situ* hybridization validate *Grp-*VGLUT1 populations in the LA, and posterior BMA; such as *Cartpt-*expressing GLUT1-14. **d**, Multiplex fluorescent *in situ* hybridization validate VGLUT2 cells in the LA, alongside *Rspo2*; such as *Penk-*expressing GLUT2-24. **c-d**, scalebars for zoom-ins, 50μm.

Many cell types of both glutamatergic classes were associated with the accessory (vomeronasal) and main olfactory systems. For example, VGLUT1 types 20–26 of the posterior COA and PAA and VGLUT2 types 27–35 of the MEA were putative accessory olfactory system neurons and shared expression of *Trh*^*+*^*Calb1*^*+*^*Zeb2*^*−*^. COA *Trh*^*+*^ amygdala neurons were described in anxiolytic behavior^50^, and recently, in suppression of male mating behavior through a COApm-MEA connection^6^. Cell types of the main olfactory system’s anterior COA, VGLUT2 types 2–8, on the other hand, were *Zeb2*^*+*^ and coexpressed *Tgfb2* and *Synpr*. These, as well as *Synpr*^*−*^*Tgfb2*^*−*^ VGLUT2 types 9–19, were located in the surprisingly molecularly diverse BMA and anterodorsal MEA, or PAA. *Fezf1* distinguished molecularly similar VGLUT2 types of the MEA (*Fezf1*^+^) from the BMA (*Fezf1*^*−*^; Fig. 3e and Extended Data Fig. 4). Six VGLUT1 types (10–15) mapped to the posterior BMA (Extended Data Fig. 6). They expressed combinations of *Meis2*, *Calb2* and *Dcn*, and *Mpped1*/*Tac1*, *Cd36*, *Gpr101*, *Cartpt* or sialyltransferase *St8sia2*, *Ptpru* and serotonin receptor *Htr2c*.

One large, *Slc17a6*–*Slc17a7* double-positive population, VGLUT1 type 8, was marked by an array of distinct genes—*Sim1*, *C1ql1*, *Pou3f1* and *Cbln2*. It was located in the NLOT (Fig. 3h and Extended Data Fig. 9), a cortical structure of the olfactory amygdala area embedded between the anterior striatal and classical cortical amygdala areas, with projections to both the main olfactory bulb (MOB) and BLA (Fig. 3i). In addition, VGLUT1 types 6 and 7 populated NLOT layer 3, but also LA and BLA-v (Extended Data Figs. 2 and 9). Slightly posterior–lateral to the NLOT was *Slc17a6*–*Slc17a7* double-positive VGLUT2 type 1 (*Trh-Tes*), which best matched with the bed nucleus of the accessory olfactory tract (BA; Extended Data Fig. 2), often considered the vomeronasal system’s equivalent of the NLOT^51^.

Related to NLOT neurons by their shared expression of *Zeb2*, *Rph3a* and *Lypd1* were BLA/LA glutamatergic cells (Fig. 3j and Extended Data Fig. 6). BLA principal cells were described in depth by their topographical distribution and circuitry (projections) and differential valance assignment, that is, their encoding of positive versus negative valance^5,11,52^. The most cell-rich branch, VGLUT1 types 1–4, expressed *Man1a*/*Dkk3* and was localized in an anterior–posterior gradient across the BLA. Of these, the largest population (VGLUT1 type 2), localized to the anterior BLA, expressed known BLA marker *Rspo2*, and specifically, *Satb1*, *Neurod6*, *Rph3a* and *Cyp26b1*. Two types (VGLUT1 types 3–4) stretched across the full BLA and were marked by *Sema5a*, *Dcn*, *Tgfb2* (3) or *Wfs1*, *Sorcs2* and *Bdnf*. We identified three VGLUT1 types (27–29) enriched to the most dorsal aspect of the BLA, the lateral nucleus (LA). The most prominent among them (VGLUT1 type 28) was distinctly marked by the neuropeptide gastrin-releasing peptide (*Grp*)*. Grp*-expressing pyramidal neurons were previously identified in the LA, where a local circuitry with *Grpr-*expressing LA interneurons influenced certain aspects of fear memory^9,53^. We found sparse, but specific expression of *Grpr* in several GABAergic interneurons, such as *Vip-*expressing GABA-50 and GABA-51, *Pvalb-*type GABA-41, and a single glutamatergic type, *Trh*-expressing VGLUT1 21 of the COA. LA-*Grp* cells shared expression of *Cyp26b1*, *Neurod6*, *Satb1* and others almost exclusively with the BLA-*Rspo2* type (VGLUT1 type 2). A single VGLUT2 type (24) showed similar spatial mapping to the LA as VGLUT1 LA types (for example, VGLUT1 type 27; Fig. 3b and Extended Data Fig. 6).

### In silico trapping highlights cued fear conditioning-activated cell types

**Fig. 4:**
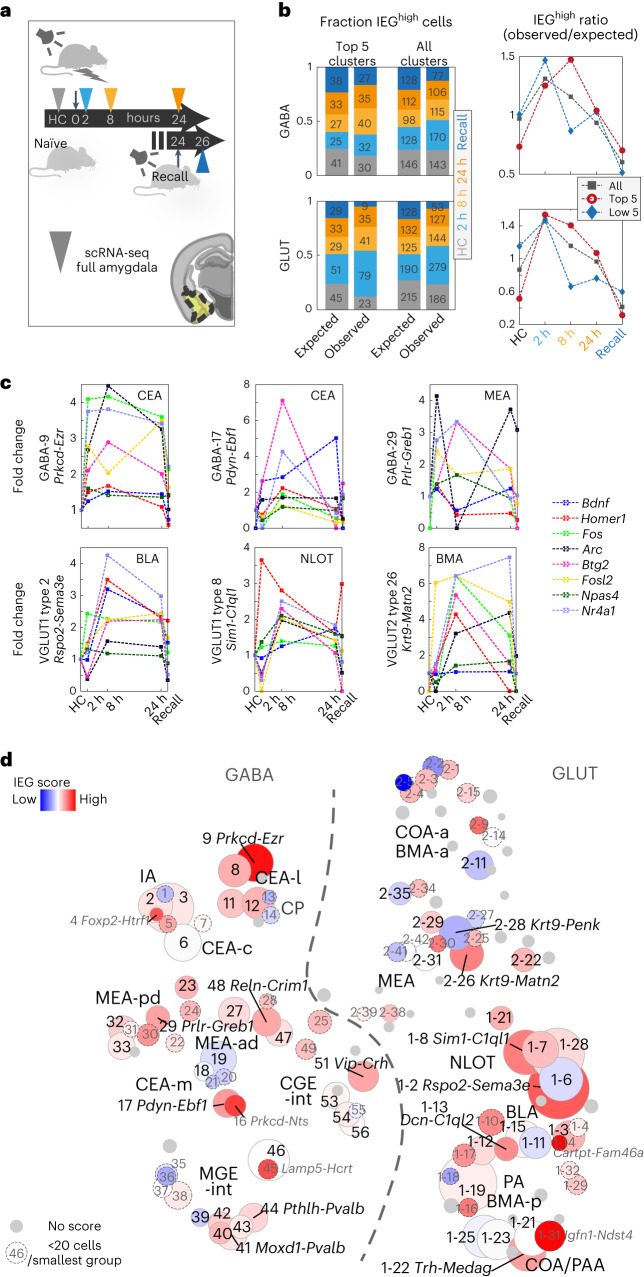
Time-resolved activity-regulated gene expression in cued fear conditioning. **a**, Tone-cued fear conditioning and scRNA-seq sampling timepoints after 2 h, 8 h, 24 h and 2 h after next-day conditioned stimulus (CS) recall (no shock). HC, naïve HC control. **b**, Per timepoint, fraction and cell number (left) of cells highly expressing IEGs *Arc*, *Bdnf*, *Btg2*, *Fos*, *Fosl2*, *Homer1*, *Npas4* or *Nr4a1* (IEG^high^, 99th percentile); observed versus the fraction and number expected by chance, shown for the five top-responding and all clusters. Right, resultant ratio (observed/expected), per timepoint. **c**, Time-dynamic expression (fold change) of the eight IEGs, 90th percentile, for the five top IEG-scoring clusters among GABA (top) and GLUT (bottom) types. **d**, Cluster-wise IEG score (Methods), visualized as heatmap on t-SNE (as in Fig. 1d). Blue, low; red, high and gray, cluster too small (*<*60 cells). Gray label/dashed circle, smallest group size <20 cells. Circle size represents cluster size. Source data

**Extended Data Fig. 7:**
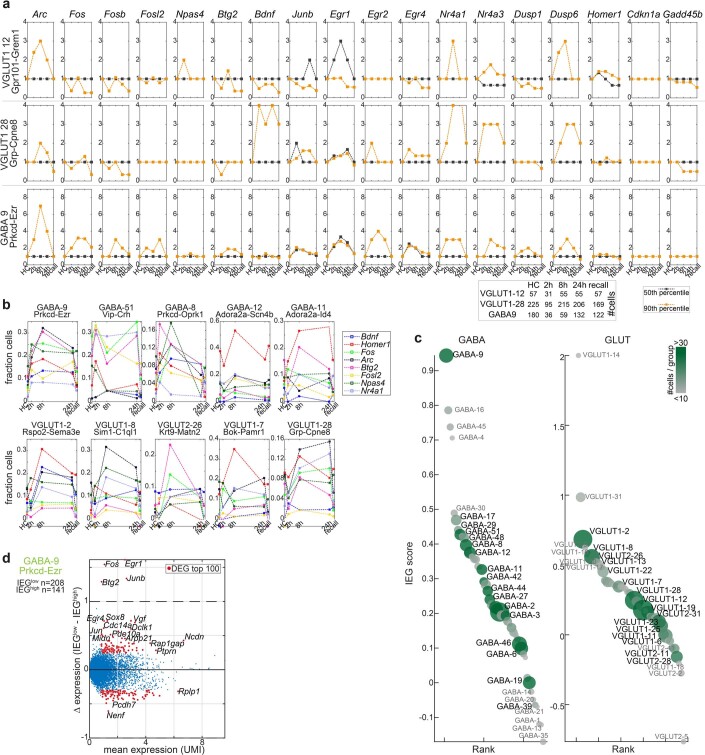
Activated cell states across the neuronal taxonomy. **a**, Time-resolved expression of IEGs for three cell types; median, black; 90^th^ percentile, orange. **b**, As Fig. 4c, in fraction cells expressing in the 90^th^ percentile, eight IEGs used for IEG score, in the five top IEG-scoring GABA and GLUT types. **c**, GABA and GLUT clusters ranked according to IEG score. Circle size represents cluster size, color represents size of smallest sampling group (gray; <10 cells, dark green; >30 cells). **d**, Scatterplot of differentially expressed genes between activated (IEG^high^, 95th percentile) and non-activated (IEG^low^) cells, for the highest IEG-scoring GLUT population. Dots represent genes, red dots; top 100 DEGs with mean UMI > 1.

Activation of neurons, for example, during memory acquisition and recall, is associated with changes in gene expression. On the transcriptional level, upregulation of activity-regulated genes, or immediate early genes (IEGs), can be detected. Activated cells within a neuronal population, based on the upregulation of one or several IEGs, are termed the memory engram. The portion of activated neurons was shown to range from as few as 2–4% after CFC in the dentate gyrus to around half, in the light-activated visual cortex^21,23,26^. We tested whether our approach could detect activated cells in any sampling timepoint after fear conditioning (context or cue), compared to the naïve home cage (HC) control group (Fig. 4a). Comparing cells from the two groups, we found no difference in their median expression of IEGs. Instead, in many cell types, we found small subsets of cells that expressed IEGs at elevated levels (for example, in the 90th percentile over all neurons; Extended Data Fig. 7). To test whether such subsets may constitute CFC-activated cell states within a cell type (the memory engram), we analyzed cells highly expressing IEGs *Arc*, *Bdnf*, *Btg2*, *Fos*, *Fosl2*, *Homer1*, *Npas4* or *Nr4a1*. Indeed, we found that activated subsets of cells were disproportionally from CFC, as compared to HC samples (Fig. 4b), and showed different time-dependent dynamics, both in fold change and fraction of activated cells (Fig. 4c and Extended Data Fig. 7).

Considering the amygdala’s heterogenous taxonomy, we then surveyed which cell types most responded to the CFC paradigm. Using the same panel of known IEGs, we summarized the fraction of activated cells, in CFC compared to HC controls, in an IEG score, calculated for each cell type (Fig. 4d and Extended Data Fig. 7). Among deeply sampled cell types ($\ge$20 neurons sampled per timepoint), the most strongly responding cell types in their respective class were the BLA *Rspo2-Sema3e* type (VGLUT1 type 2) and the CEA *Prkcd-Ezr* type (GABA-9), as measured by their IEG score. Other transcriptionally activated glutamatergic cell types were from the BMA/BLA (VGLUT1 type 13 *Dcn-C1ql2*, VGLUT1 type 3 *Sema5a-Dcn* and VGLUT1 type 12 *Gpr101-Grem1*), BMA (VGLUT2 type 26 *Krt9-Matn2* and VGLUT2 type 29 *Tac1-Cartpt*), nucleus of the olfactory tract (NLOT, VGLUT1 type 8 *Sim1-C1ql1* and VGLUT1 type 7 *Bok-Pamr1*), COA (VGLUT1 type 22 *Trh-Medag*) and LA (VGLUT1 type 28 *Grp-Cpne8*). CEA types GABA-17 *Pdyn-Ebf1* and GABA-8 *Prkcd-Oprk1*, MEA types GABA-29 *Prlr-Greb1* and interneuron types GABA-51 *Vip-Crh* and 48 *Reln-Crim1* were most activated in the GABAergic class.

On the other hand, cell types least responding with an upregulation of classical IEGs in response to CFC included several cell types from the very same amygdala subregions, such as the MEA neurons VGLUT2 type 28 *Krt9-Penk*, VGLUT2 type 11 *Ebf1-Fmod* VGLUT1 type 6 *Thrsp-Lamp5* of the BLA and VGLUT1 type 11 *Cd36-Calb2*. Similarly, among GABAergic neurons, several CEA types did not deploy cells to the engram (GABA-19 *Isl1-Aldoc*, 18 *Isl1-Tac1* and GABA-6 *Pax6-Th*), and interneurons GABA-39 *Sst-Nek7* and 46 *Lamp5-Kit* were similarly unresponsive.

Many smaller clusters of cell types (>60 cells total, <20 cells per timepoint; gray in Fig. 4d) likely also responded, although statistical confidence in their analysis is reduced. Here some notable examples of activated cell types were GABAergic ITCs GABA-4 *Foxp2-Htr1f*, compared to the nonactivated ITC population GABA-1 *Foxp2-Fmod*. Other CEA types also scored highly (GABA-16 *Prkcd-Nts* and GABA-5 *Pax6-Enpp2*), as did interneuron type GABA-45 *Lamp5-Hcrtr2*. Among smaller glutamatergic populations, BMA type VGLUT1 type 14 *Cartpt-Fam46a* stood out. Further, both ventral hippocampus (vHPF) populations, VGLUT1 type 16 *Fbln1-Il33* and VGLUT1 type 17 *Eps8-Cd44*, were among the most activated cell types. This is in line with the vHPF’s involvement in emotional behaviors, including learned anxiety and fear memory^54–56^.

### CFC-activated neurons upregulate synaptic processes

**Fig. 5:**
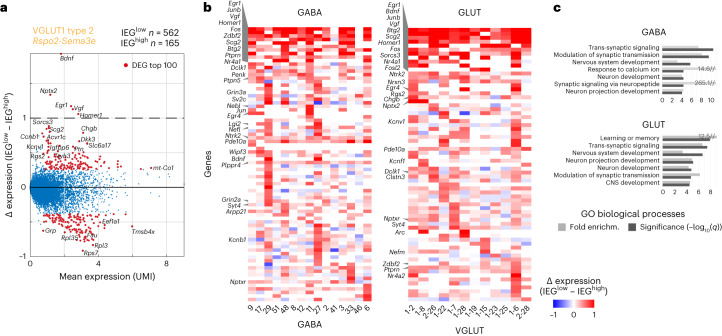
Gene expression in IEG^high^ engram cells. **a**, Scatterplot of differentially expressed genes between activated (IEG^high^, 95th percentile) and nonactivated (IEG^low^) cells, for the highest IEG-scoring GLUT population. Dots represent genes, red dots: top 100 DEGs with mean UMI *>* 1. **b**, DEGs frequently upregulated in activated (IEG^high^) cells (as in **a**), per cluster. Blue, low and red, high. **c**, Biological processes enriched in activated (IEG^high^) cells (as in **a**) per class; FDR-adjusted *q* values, and fold enrichment (DAVID). Source data

**Extended Data Fig. 8:**
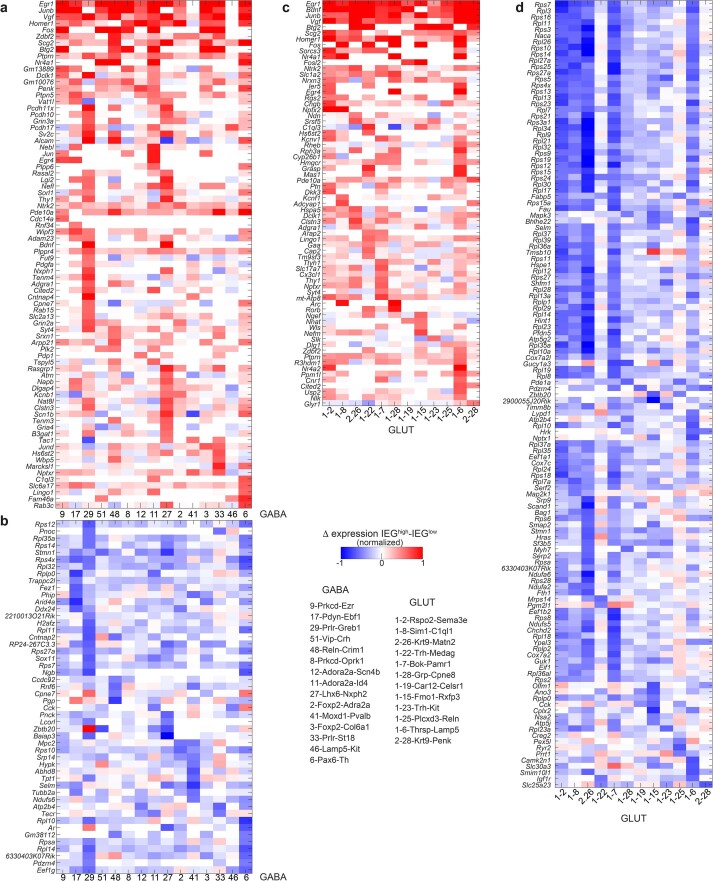
Differential gene expression in IEG^high^ cells. Heatmaps of upregulated (**a**,**c**) and downregulated (**b**,**d**) genes in IEG^high^ cells, per cell type, large clusters only. Cluster order by IEG score. Blue, low; red, high expression. Source data

Next, we analyzed which other genes covaried with the IEG panel; for each cell type, we analyzed what distinguished activated from nonactivated cells. We used the eight-IEG panel (*Arc*, *Bdnf*, *Btg2*, *Fos*, *Fosl2*, *Homer1*, *Npas4* and *Nr4a1*) to ‘trap’ single cells expressing any IEG in the 95th percentile over all neurons (activated cells). We then analyzed differential gene expression between this activated population (IEG^high^) versus nonactivated cells of the same cell type (IEG^low^; Fig. 5a and Extended Data Fig. 8). Across all cell types, besides the genes of the defined IEG panel (the ‘trap’), other known IEGs were also upregulated, such as *Egr1* (or Zif268), *Egr4*, *Junb or Scg2* (Fig. 5b). Globally, biological processes upregulated in association with the activated state were related to synaptic signaling and modulation of synaptic transmission/plasticity, but also development, projection morphogenesis, and learning and memory (Fig. 5c). For example, *Ntrk2* and *Vgf* are well-documented actors in synaptic plasticity and CFC acquisition^57,58^ that showed robust, activity-dependent transcriptional upregulation. *Ntrk2* (TrkB) encodes a receptor for neurotrophins BDNF and VGF, both of which we also found upregulated in activity-induced neurons. Activated TrkB affects neurite outgrowth and synapse formation and plasticity via phosphorylation of CREB. We found many other robust candidates that were shared across activated cells in GABAergic and glutamatergic types, such as *Dclk1*, *Syt4*, *Clstn3*, *Pde10a*, *Ptprn and Zdbf2*.

Other upregulated genes were less widely studied. Neuronal dense core vesicle component synaptotagmin 4 (*Syt4*) is involved in dendrite extension and phosphatase *Ptprn* in vesicle-mediated secretory processes. Phosphodiesterase *Pde10a* regulates the intracellular concentration of cyclic nucleotides and was shown to be upregulated after LTP induction^59^. Pentraxin receptor *Nptxr* participates in synapse remodeling, and *Clstn3* is a regulator of synapse assembly^60^. Processes related to translation on the other hand were downregulated in activated cells (via, for example, ribosomal protein genes; Extended Data Fig. 8).

In addition, some class-specific differences were apparent (Fig. 5b). For example, activated GABAergic cells specifically upregulated *Arpp21* that was shown to be essential in dendritic branching and complexity^61^, synaptic vesicle protein *Sv2c* and cytoskeleton-related transcripts *Tiparp* and *Wipf3*. GABA-specific activity-induced phosphatase *Ptpn5* (also known as STEP) regulates several effector molecules involved in synaptic plasticity, *Plppr4* is important for axonal outgrowth during development and regenerative sprouting, and attenuates phospholipid-induced axon collapse. Although not exclusive, *Bdnf* was more widely induced in glutamatergic cells. Specific to activated glutamatergic cells were neuropeptide receptor *Sorcs3*, granin *Chgb* and synaptic proteins *Nrxn3* and *Nptx2*, the latter implied in long-term plasticity. Voltage-gated potassium channels *Kcnv1* and *Kcnf1* were also enriched in activated glutamatergic cells.

Some cell-type-specific patterns of activation also emerged—in activated cells of several GABAergic types of the medial amygdala (GABA-27, GABA-29 and GABA-33), inhibitory synapse specifier *Lgi2* (ref. ^62^) was highly expressed. *Egr4*, on the other hand, was restricted to activated cell types of the CEA (GABA-9 and GABA-11), BLA and LA (VGLUT1 types 2, 6 and 28; Fig. 5b).

### CFC-induced correlation of learning gene modules

**Fig. 6:**
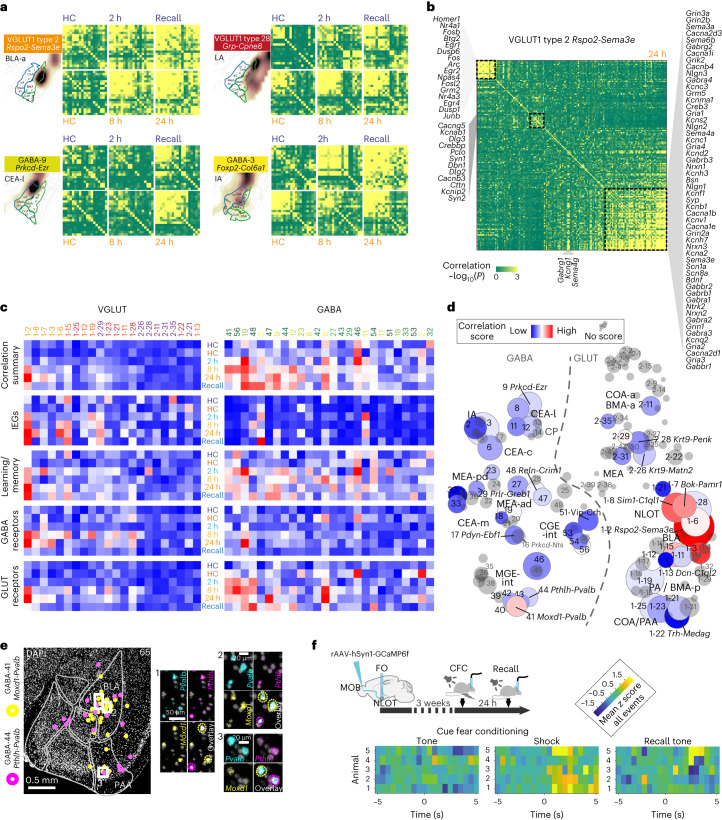
Gene expression correlation identifies cell types with CFC-activated learning gene modules. **a**, Pearson pairwise correlation (green, low and yellow, high) of 18 IEGs for four cell types, resolved by post-CFC sampling timepoint and batch-specific HC control. For each cell type; top row, batch B (HC-2 h-recall); bottom row, batch A (HC-8 h-24 h); genes ordered by hierarchical clustering. **b**, Pairwise correlation of 156 learning-related genes, in VGLUT1 type 2, 24 h post CFC, with three correlated gene expression modules highlighted. Pearson coefficient (green, low and yellow, high), genes are ordered by hierarchical clustering. **c**, Time-resolved correlation, per experimental group (post-CFC, HC controls), for large clusters (smallest group in cluster $\ge$20 cells), for gene modules indicated, or all genes combined (‘correlation summary’). **d**, CFC-induced correlation across the full amygdala taxonomy, visualized as heatmap on t-SNE (as in Fig. 1b). Pairwise correlation score (gray, no score (smallest group in cluster <20 cells)). Circle size represents cluster size. **c**,**d**, Pearson pairwise correlation score (blue, low and red, high). **e**, Multiplex fluorescent in situ hybridization validates two *Pvalb*^*+*^ interneuron populations GABA-41 and GABA-44 (with different transcriptional responses to CFC) had similar spatial distributions primarily in the BLA. **f**, Targeting strategy for validation of NLOT participation in CFC via retrograde labeling of MOB-projecting neurons with GCaMP6f, and fiberphotometry recording in NLOT, during CFC and recall. Mean *z* score of NLOT calcium activity recorded in seven CFC tone-shock pairings (left), and four no-shock tone recall events (right), visualized as heatmap 5 seconds before and after the event, for five mice (rows). Source data

**Extended Data Fig. 9:**
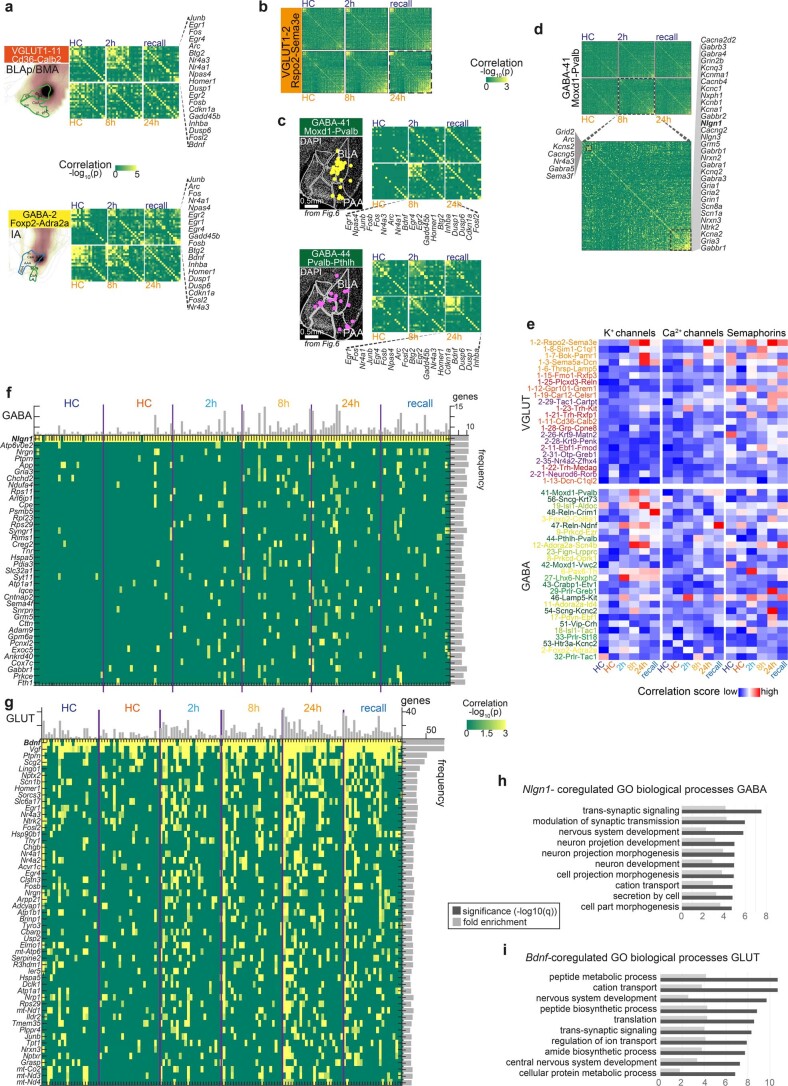
Gene expression correlation as a response to CFC. **a**, Pearson pairwise correlation coefficient (green, low; yellow, high) of 18 IEGs (rows and columns) for two cell types, resolved by post-CFC sampling time point and batch-specific home cage (HC) control. For each cell type; top row, batch B (HC-2h-recall); bottom row, batch A (HC-8h-24h); genes are ordered by hierarchical clustering, indicated on the right. **b**, For VGLUT1 2 Rspo2-Sema3e, pairwise correlation of 155 learning-related genes (rows and columns), per post-CFC sampling time point and batch-specific home cage (HC) control. Pearson coefficient (green, low; yellow, high), genes are ordered by hierarchical clustering. **c**, Pearson coefficient (green, low; yellow, high) of 18 IEGs (rows and columns) for Pvalb-interneuron types GABA 41 and -44, resolved by post-CFC sampling time point and batch-specific home cage (HC) control. For each cell type; top row, batch B (HC-2h-recall); bottom row, batch A (HC-8h-24h); genes are ordered by hierarchical clustering, indicated below. Left, distribution of each population in one amygdala section by multiplex *FISH* (see Fig. 5e) **d**, For GABAergic interneuron type Moxd1-Pvalb, pairwise correlation of 155 learning-related genes (rows and columns), per post-CFC sampling time point and batch-specific home cage (HC) control. Pearson coefficient (green, low; yellow, high), genes are ordered by hierarchical clustering. Zoom-in to 8 h post-CFC (right), with two correlated gene expression modules highlighted. **e**, As Fig. 6c; time-resolved correlation, per experimental group (post-CFC, HC controls), for large clusters (smallest group in cluster $\ge$20cells), resolved for sampling timepoints and batch-specific home cage (HC) controls, for gene modules indicated. **f**,**g**, For large clusters, Pearson coefficient for genes highly and frequently correlated with neuroligin *Nlgn1* in GABA (**f**) or *Bdnf* in GLUT (**g**), per sample group (timepoints post-CFC, and naïve home cage (HC) per batch (orange, batch A; blue, batch B). Green, low; yellow, high. Rows, genes; columns, cell types (ordered by overall correlation score, as **e**). Bars on the top and left are the sum of each column or row (p > 0.01), respectively. **h**,**i**, Biological processes enriched among the top 200-genes correlated with *Bdnf* (GLUT) or *Nlgn1* (GABA); FDR-adjusted q-values, and fold enrichment (DAVID). Source data

Finally, we investigated to what extent IEGs were coexpressed in individual cells, rather than just individually highly expressed (IEG^high^). To this end, we quantified pairwise correlation of IEG expression, per cell type, and experimental group. Like IEG activation, IEG correlation, too, was restricted to a subset of all neuronal populations in the amygdala, and was consistently greater after CFC, compared to HC controls. Cell types with high IEG-activation scores often, but not always, had high IEG correlation, too. For example, IEGs in the activity high-scoring GABAergic population, *Prkcd-Ezr*, were particularly highly correlated 24 h after CFC and 2 h after recall. And while IA types *Foxp2-Adra2a* and *Foxp2-Col6a2* (GABA-2 and GABA-3) showed similar IEG activity scores (Fig. 4d), their correlation dynamics differed vastly; with only *Foxp2-Col6a2* strongly coregulating IEGs, particularly in the later sampling timepoints, and recall (Fig. 6a and Extended Data Fig. 9).

We further explored whether other known learning-related genes, receptors and channels (Supplementary Table 4) displayed similar correlation dynamics. Indeed, we found an increased correlation in a subset of cell types, and considerable differences between experimental groups (Fig. 6b–d and Extended Data Fig. 9). For example, VGLUT1 2 *Rspo2-Sema3e* showed strongly correlated, time-dynamic expression across all analyzed gene modules (Fig. 6b), while other BLA/LA clusters had more subtle responses (VGLUT1 11, VGLUT1 28), or lacked any CFC-induced coregulation (VGLUT1 13). Among GABAergic cells, the BLA-*Moxd1-Pvalb* interneuron GABA-41 scored highly in correlation with nearly all learning-related genes after CFC, while the panel of IEGs showed no correlation (Fig. 6c and Extended Data Fig. 9). This pattern was evident for most GABAergic clusters, such as a lower-scoring, closely related BLA-*Pthlh-Pvalb* population (Fig. 6c–e), and suggests a different transcriptional dynamic or regulation in GABAergic, compared to glutamatergic cells.

### Coregulation characterizes cellular events after memory retrieval

Among many analyzed genes, *Bdnf* (in GLUT) and *Nlgn1* (in GABA) were examples we frequently found highly correlated with both IEGs and other learning-related modules. To examine to what extent they may represent the wider orchestrated, transcriptional response to CFC, we analyzed each of their correlation, with transcriptome-wide gene expression (Extended Data Fig. 9). For both, correlation strength and frequency greatly increased after CFC, compared to HC controls. *Bdnf* most strongly correlated with co-TrkB ligand *Vgf*, and several of the same genes highly expressed in IEG^high^ cells (Extended Data Fig. 8); for example, *Ptprn*, *Nptx2*, *Scg2*, *Lingo1* or *Scn1b*. *Nlgn1* correlation among GABAergic clusters revealed several different candidates, such as synapse-acidifying proton pump *Atp6v0e2*, neurogranin *Nrgn* and amyloid β precursor *App*. Enriched biological processes concerned ion transport, *trans*-synaptic signaling, development and translation, peptide metabolism and biosynthesis (Extended Data Fig. 8).

We used IEGs to identify engram cells because optogenetic and pharmacogenetic studies have shown that IEG-expressing neurons are required for memory recall. Transcriptional changes occurring in direct response to a memory recall event, however, have been described to a much lesser extent. For example, *Egr1* (Zif268) levels were reported to change specifically after memory retrieval^63^. In our data, *Egr1* was not specific to recall, but highly upregulated, with other IEGs, in all activated cell types. To the contrary, we found that the very IEGs that initiate processes required for memory acquisition were not elevated 2 h after recall. Instead, IEG expression appeared less active—both fraction of IEG^high^ cells and fold change in their IEG expression were lower after recall than all other timepoints, including naïve HC controls, and the temporally close 24 h post-CFC samples (Fig. 4b and Extended Data Fig. 8). Analysis of differentially expressed genes (DEGs) between same-batch recall and 2 h only confirmed that cells expressed consistently higher levels of IEGs 2 h post-CFC than 2 h after CS recall. Here no other biological processes or pathways were substantially enriched for either condition (Supplementary Table 5).

In our analysis of correlated genes, however, recall often showed highly coregulated expression, across gene modules and cell types. Even IEGs, although lower expressed, were in many cases more correlated (that is, more likely to be expressed in the same cell) 2 h after recall than 2 h after CFC. In summary, more orchestrated gene expression, rather than elevated IEG expression, may describe cellular processes associated with memory consolidation and retrieval.

### Ambivalent functional response in the NLOT

**Extended Data Fig. 10:**
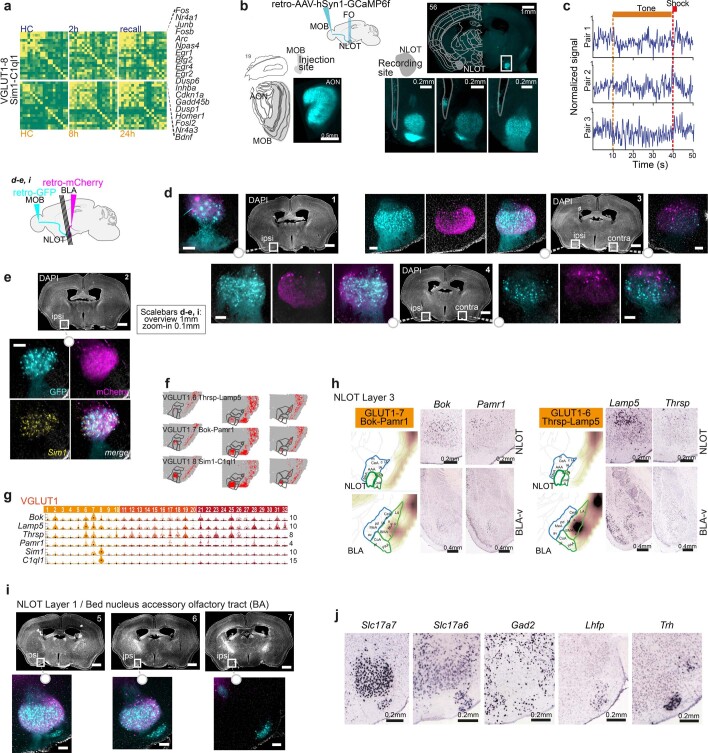
NLOT’s participation in CFC, and diversity in molecular NLOT types and projections. **a**, Pearson pairwise correlation (green, low; yellow, high) of 18 IEGs (rows and columns) for VGLUT1-8, resolved by post-CFC sampling time point and batch-specific home cage (HC) control. Top row, batch B (HC-2h-recall); bottom row, batch A (HC-8h-24h); genes are ordered by hierarchical clustering, indicated on the right. **b**, Expression of retro-AAV-hSyn1-GCaMP6f (cyan) in the injection site (left; MOB, with accessory olfactory nucleus (AON) strongly labeled), and recording site (right; NLOT) of one mouse. NLOT zoom-ins, 3 a.-p. positions reveal GCaMP6f expression in layer 1 and 2 NLOT, and tissue damage (outlined) indicates proximal optic-fiber tip placement. **c**, Examples of individual calcium-traces in three consecutive tone-shock pairings; with tone and shock duration indicated. **d**,**e**, NLOT projection tracing by injection of retro-AAV-hSyn1 to MOB (GFP; cyan) and BLA (mCherry; magenta). MOB-projecting NLOT neurons (cyan) primarily populate ipsi- and contralateral NLOT layer 2, BLA-projecting NLOT neurons (magenta) dominate layer 3; both populations partially express *Sim1* (**e**). **f**, Spatial correlation to three Visium ST sections, for NLOT populations VGLUT1 6-8. **g**, Gene expression visualized as single-cell violin plots. **h**, Two additional populations sparsely label NLOT layer 3, but also BLA-v; indicated by inferred spatial distributions (AMBA volumetric correlation), and individual marker genes in Allen Mouse Brain Atlas (*Bok*, https://mouse.brain-map.org/experiment/show/71064032; *Pamr1*, https://mouse.brain-map.org/experiment/show/70744322; *Lamp5*, https://mouse.brain-map.org/experiment/show/70927827; *Thrsp*, https://mouse.brain-map.org/experiment/show/71587845). **i**, NLOT projection tracing (as **d**,**e**) in posterior sections reveal MOB-projecting (but not BLA-projecting) NLOT neurons (cyan) also populate NLOT layer 1, and putative bed nucleus of the accessory olfactory tract (BA). **j**, Individual genes expressed in NLOT layer 1 and BA (bed nucleus of the accessory olfactory tract), from Allen Mouse Brain Atlas (*Slc17a7*, https://mouse.brain-map.org/experiment/show/70436317; *Slc17a6*, https://mouse.brain-map.org/experiment/show/73818754; *Gad2*, https://mouse.brain-map.org/experiment/show/79591669; *Lhfp*, https://mouse.brain-map.org/experiment/show/73769323; *Trh*, https://mouse.brain-map.org/experiment/show/71016631).

The NLOT population VGLUT1 8 *Sim1-C1ql1* contained a large fraction of IEG^high^ cells (Fig. 4), and also showed orchestrated induction of learning-related gene modules, especially in the later timepoints (8 h and 24 h) and after recall (Fig. 6c,d and Extended Data Fig. 9). To examine whether this nucleus has a role in CFC, and to what extent gene expression may correspond to physiological events, we injected retroAAV-GCaMP6f to the MOB of eight adult males and measured the NLOT’s response to cued fear conditioning, and recall, 3–4 weeks post surgery, using fiber photometry (Fig. 6f). Five of the eight mice showed GCaMP6f expression in NLOT and fiber placement proximal to the NLOT (Extended Data Fig. 10). The measured calcium activity was mild but consistent; over 7 CFC-pairings, we found increased signal immediately after the shock (paired *t*-test; *T*_1,4_ = 2.741, *P* < 0.01), but not tone (*P* > 0.5). The next day, four recall events (no shock) resulted in calcium activity changes that were inconsistent between individuals (Fig. 6f; *P* > 0.5). We tested whether this mixed response may be due to the remaining heterogeneity of the targeted population by examining its projections and overlap with *Sim1* expression. While MOB-projecting NLOT neurons mostly expressed *Sim1* in layer 2, a *Sim1-* population in NLOT layer 1 was likely recorded using our targeting strategy (Extended Data Fig. 10). Further, BLA-projecting NLOT neurons partially expressed *Sim1*, but preferentially populated NLOT layer 3, and likely made up a mix of populations (VGLUT1 6–8; Extended Data Fig. 10). Together, functional interrogation of cell types requires molecular resolution, but also consideration of projection patterns.

## Discussion

We present a taxonomy of mouse amygdala cell types and reveal molecular details over this region’s functionally distinct and intertwined subregions. In this complex spatial and molecular context, we present a taxonomy-wide analysis of transcriptional response in the hours following fear learning and memory recall. The study highlights amygdala cell types with activated transcriptional states and genes with potential roles in learning and memory. We argue that single-cell resolution and unbiased, transcriptome-wide coverage are critical in identifying the response that is expected in only a small subset of cells (the engram) of particular cell types.

We applied two approaches using the high dimensionality of scRNA-seq. First, we compared activated versus nonactivated cell states per cell type. Essentially, this mimicked the experimental use of mouse models using fluorescent reporters to identify IEG-expressing engram neurons^64,65^ (for example, *Arc::dVenus*) in silico. We found, however, that a single IEG was unlikely to mark the full pallet of neuronal activation at every timepoint and we opted to use a consensus panel of activity-regulated genes (IEGs) instead. Indeed, different IEGs were highly expressed in just a subset of neurons and displayed time-dependent dynamics in response to fear conditioning. This observation was in line with activity-regulated genes that expanded well into consolidation in another transcriptomics study^26^. Importantly, the IEG response was restricted to a subset of the taxonomy’s cell types, and within those, only a subset of cells strongly upregulated IEGs—consistent with a sparse engram encoding any particular memory^1^. In our second approach, we present gene expression correlation as a powerful tool to identify subsets of cells within cell types that display orchestrated responses related to learning and memory, especially when IEG induction is absent (recall). We identified gene signatures that were regulated in activated subsets within each cell type, confirmed known actors, and described new candidate genes with some class specificity. For example, *Bdnf* and *Nlgn1* could serve as predictors for the induction of learning-related cellular processes in similar datasets. Activated engram cells upregulated and coregulated activity-response genes, and processes of synaptic signaling, plasticity, development and neurite outgrowth.

Across the amygdala’s taxonomy, closely related molecular cell types from the same anatomical subregion could have a strong response or lack activation. This observation may help explain the encoding of opposing valences (for example, appetitive versus aversive)^19^ in cells and circuitries of the same subregion. For example, among regions studied in associative learning, such as fear or appetitive conditioning, BLA glutamatergic cells were greatly diverse and, by the transcriptional response measured in our study, indicated strong (for example, VGLUT1 type 2) or limited to no involvement (for example, VGLUT1 types 11 and 13) in CFC. In addition, their molecular identities correlated with different anterior–posterior distributions, which could link to previous findings that BLA principal cells along the anterior–posterior axis encode opposing valences^5,11,52^. Indeed, *Rspo2*^*+*^ cells (VGLUT1 type 2), located anteriorly within the BLA, were consistently a highly activated population, while *Dcn*^*+*^ and *Cd36*^*+*^ in the posterior BLA and BMA (VGLUT1 types 13 and 11) tended to score lower in response to CFC. Similarly, the BLA’s downstream target, CEA GABAergic cells, had two highly responsive *Prkcd*^*+*^ subtypes (GABA-9 and GABA-16).

Beyond these activated BLA and CEA populations that encouragingly resemble cells characterized in their participation in memory engrams using functional and physiological methods, our study also highlighted other cell types transcriptionally affected after cued fear learning and recall. Their detailed molecular description will ease future exploration for their precise roles as potential players in CFC, or other behavioral paradigms. For example, we validated a small population of *Cartpt*^*+*^ neurons in the BMA (VGLUT1 14), which strongly induced IEG expression as a response to CFC, and two highly related *Krt*^*+*^ VGLUT2 populations, VGLUT2 type 26 (MEA) and 28 (BMA), responded at greatly different intensities to CFC learning and recall. In line with functional studies^66–68^, several interneuron types responded to CFC. They were marked by induced correlation of learning-related genes, rather than upregulation of IEGs only—BLA *Pvalb*^*+*^ interneurons (with a *Moxd1*^*+*^ more than a *Pthlh*^*+*^ subtype), but also one *Snc*^*+*^, and two *Reln*^*+*^ populations.

In the NLOT, we identified and validated a large population of molecularly distinct *Sim1-C1ql1* neurons (VGLUT1 8) that may be involved in associating context-based odor information with behavior^69,70^. This population was highly responsive both in the proportion of IEG^high^ cells after CFC and induced correlation of learning and memory-related genes. Fiber photometry recordings of MOB-projecting NLOT neurons’ calcium response were inconclusive. The population responded to shock, but no consistent response was evident during recall. This may of course be because the NLOT does not participate in CFC recall, or because calcium activity does not parallel the CFC response observed using scRNA-seq. Further, we discovered remaining heterogeneity in the NLOT’s cell types, layer composition and projections. This emphasizes how functional studies need to ideally consider detailed molecular knowledge and/or projection patterns when targeting specific neuronal populations, as was recently demonstrated in the CEA^19,20^.

Single-cell transcriptomics was used for detecting transcriptional signatures of neuronal activation^21,22^. It has been suggested and gained momentum as a tool for comprehensive, unbiased identification of cellular and molecular substrates of learning and memory^26,27,71^. As a word of caution, however, the approach and our study contain a few limitations. First, even highly refined current protocols dependent on the physical dissociation of live cells may suffer from dissociation stress and sampling noise. Second, identifying subtle transcriptional states at cell-type resolution requires deep sampling, making scRNA-seq an expensive approach, especially when considering several experimental groups, appropriate controls and biological repeats.

We provide a browsable companion website of cluster-wise scRNA-seq gene expression and spatial correlation data that may guide study design. We expect that increasingly highly sensitive, high-dimensional ST methods with cellular resolution (for example, MERFISH) could fine-map cell types, but also detect panels of learning-related genes, and perhaps even single-cell resolved transcriptional correlation. In addition, it will be of increasing interest to consider how a cell type’s connectivity affects its involvement in any particular function—as it remains to be shown if high-quality transcriptional signatures could sufficiently predict cell type, cell state and connectivity, as cell-type atlases evolve. Given the plethora of available behavioral paradigms and genetic tools, this taxonomy may serve to clarify the particular contributions of genes, and molecularly defined neurons in learning and memory, and other emotional and social behaviors.

## Methods

### Mice and ethics

We used adult C57Bl/6J wildtype mice, 7–12 weeks old; CFC and scRNA-seq, 22 males and 1 female; Visium ST, 1 male and 1 female; retrograde viral tracing (MOB, BLA), 4 females; and fiber photometry, 8 males. Most mice were of the C57Bl/6JOlaHsd subline (Envigo) and therefore carried a known α-synuclein (*Snca*) deletion previously evaluated with respect to fear conditioning^72^. Mice used for scRNA-seq and their corresponding experimental groups are listed in Supplementary Table 1. Mice were group housed at standard conditions of 21–23 °C and 30–40% relative humidity, under a reversed day–night cycle, and provided standard chow and water ad libitum. All experiments were carried out during the night cycle. All experimental procedures followed the legislation under the National Institute of Health guidelines and were approved by the Institutional Animal Care and Use Committees of the Technion Israel Institute of Technology and the University of Haifa.

### Tone-cued fear conditioning

Seven to 11-week-old male C57Bl/6JOlaHsd mice were habituated to a behavioral arena for 20 min, followed by six cycles of US–CS pairing (cued fear conditioning, CFC)—35 s tone (CS), 750 ms 0.7 mV foot shock (US) and 24 s rest. US–CS association was quickly and robustly established, characterized by freezing behavior during tone cue, typically after the second cycle (data not shown). For learning and consolidation, mice were sampled 2 h (*n* = 5), 8 h (*n* = 4) and 24 h (*n* = 4) after CFC. For recall, 24 h after CFC, four mice were exposed to six cycles of the tone cue alone (35 s CS and 25 s rest). The three mice exhibiting the strongest freezing behavior compared to non-FC controls undergoing the same session (video quantified, data not shown) were killed 2 h later. Naïve, HC littermates served as controls in each experimental batch (*n* = 7).

The data used for analysis of fear conditioning were collected in the following two experimental batches (Supplementary Table 1): batch A was collected to examine consolidation (8 h (*n* = 4) and 24 h (*n* = 4) post-CFC versus naïve (*n* = 2)), while in batch B, we investigated earlier responses, either 2 h after CFC (*n* = 2) or 2 h after CS recall (where CS recall was 24 h after CFC, *n* = 3) versus naïve (*n* = 2). The remaining samples were used for the cell-type taxonomy only. We detected all cell types in all sampled mice, both naïve- and fear-conditioned, at ratios expected according to the sampling depth (Extended Data Fig. 1).

### Perfusion

Mice were killed by an overdose of ketamine/medetomidine, followed by transcardial perfusion with freshly prepared, ice-cold, carboxygenated NMDG-based artificial cerebrospinal fluid (aCSF; 93 mM NMDG, 2.5 mM KCl, 1.2 mM NaH_2_PO_4_, 30 mM NaHCO_3_, 20 mM HEPES, 25 mM d-glucose, 5 mM Na-ascorbate, 2 mM thiourea, 3 mM Na-pyruvate, 10 mM MgSO_4_ and 0.5 mM CaCl_2_, adjusted to pH 7.3–7.4 with concentrated HCl)^73^. Brains were quickly removed and maintained on ice in aCSF.

### Amygdala dissection

Freshly aCSF-perfused brains were quickly mounted on a Leica VT1200S Automated Vibrating Microtome and sectioned to 300 μm coronal slices. Sections were then quickly microdissected in cold aCSF, to sample the full amygdala, according to the following anatomical enclosures: for mediolateral reference, we used hypothalamus and optic tract medially, and laterally, a notional ventral extension of the amygdalar capsule of the corpus callosum fiber tracts. The beginning and end of the bifurcation of the external and amygdalar capsules marked visible anterior–posterior amygdala sampling borders. Microdissections were performed but as anatomically precise and reproducible as possible.

### Cell suspensions and scRNA-seq

Microdissected tissue pieces were digested in 800–1,000 μl papain digest solution per amygdala (Worthington Papain system, vial 2 reconstituted in 5 ml aCSF, and 5% DNase (vial 3 reconstituted in 500 μl aCSF)), 25–30 min at 37 °C, until mechanical trituration with a wide-diameter fire-polished glass pipette easily separated most of the tissue. Next, the remaining tougher vascular or ventricular pieces were removed by filtering the digested suspension through an aCSF-equilibrated 30 μm cell strainer (Partec CellTrix), to a BSA-coated microcentrifuge tube. Cells were pelleted at 200*g* for 5 min at 4 °C and resuspended in 200 μl aCSF with 2.5% DNaseI (Worthington Papain system, vial 3 reconstituted in 500 μl aCSF). For myelin and debris removal, the suspension was layered on top of 1 ml 5% OptiPrep (Sigma-Aldrich) in aCSF in a BSA-coated microcentrifuge tube, and centrifuged at 150*g* for 6 min at 4 °C, with slow ramping. The resulting cell pellet was resuspended in a minimal volume of aCSF. Cells were inspected in a Burker counting chamber for intact, bright, nongranular cell morphologies, indicating high viability and successful debris removal. At all steps, from perfusion to final single-cell suspension, tissue or cells were maintained in ice-cold carboxygenated (95% O_2_, 5% CO_2_) aCSF—with the exception of papain digest, where the temperature was 37 °C.

Single-cell suspensions were diluted to 1,000 cells per μl, and processed for 10×Chromium-v3 GEM generation and scRNA-seq. We followed the manufacturer’s instructions, targeting 5,000–6,000 cells per sample. Sequencing libraries were multiplexed and sequenced on Illumina NextSeq or NovaSeq NGS platforms, targeting a depth of >35–40K reads per cell.

### Quantification and statistical analysis of scRNA-seq data

To interpret our scRNA-seq data for the purpose of cell-type discovery, we largely followed the logic of our tested-and-proven analysis pipeline, detailed in ref. ^14^. First, raw sequencing data were demultiplexed, aligned with the genome, and mRNA molecules counted on the cell ranger pipeline (10x Genomics), resulting in output matrix UMI count files. Next, we conducted an informal exploratory analysis and found robust division of cells into non-neuronal and three main neuronal classes (GABA, VGLUT1 and VGLUT2), which led us to apply the following iterative clustering approach.

#### Iterative clustering

All final analysis was performed in our MATLAB-based clustering pipeline (described in the next section), in the following three iterations:

##### Step 1: cell QC and classification

To extract and classify neurons, we set the threshold of 3,000 UMI per cell and 2,500 genes per cell, then followed standard pipeline steps (see below) to obtain rough clusters that were classified into four categories (GABA, VGLUT1, VGLUT2 and non-neuronal) based on a majority vote of known markers (*Gad2* = GABA, *Slc17a7* = VGLUT1, *Slc17a6* = VGLUT2 and non-neuronal cells (which were excluded) = *C1qc* *+* *C1qa* *+* *C1qb* *+* *Gja1* *+* *Cx3cr1* *+* *Acta* + *Ly6c1* *+* *Mfge8* *+* *Plxnb3* *+* *Cldn11* *+* *Aqp4* *+* *Vtn* *+* *Cldn5* *+* *Pdgfrb* *+* *Flt1* *+* *Slc25a18* *+* *Pdgfra* *+* *Foxj1* *+* *Olig1* *+* *Olig2* *+* *Sox10* *+* *Hbb-bs* *+* *Hbb-bt* *+* *Hba-a2* *+* *Ttr*). Doublets were called and excluded at this point if a cluster combined any of the above gene sets at a ratio <2, except the permitted combination of VGLUT1 and VGLUT2.

To identify and classify non-neuronal cells, we set a lower threshold of 2,000 UMI per cell and 1,000 genes per cell, and excluded cells that expressed either *Gad2*, *Slc17a7* or *Slc17a6*. Non-neuronal clusters were classified into the following categories: immune (based on *C1qc*, *C1qa*, *C1qb*, *Mrc1*, *Pf4* and *Cx3cr1*), astrocytes (based on *Gja1*, *Aqp4*, *Foxj1*, *Aldoc, Mfge8* and *Slc25a18*), vascular (based on *Vtn*, *Cldn5*, *Pdgfrb*, *Flt1*, *Acta2* and *Ly6c1*), oligodendrocytes (based on *Plxnb3*, *Cldn11*, *Olig1*, *Olig2*, *Sox10* and *Pdgfra*) and blood (which were excluded, based on *Hbb-bs*, *Hbb-bt*, *Hba-a2* and *Ttr*).

##### Step 2: cluster calling, QC and doublets removal

For retained cells of each Step 1 category (GABA, VGLUT1, VGLUT2, immune, astrocytes, vascular and oligodendrocytes), we reran the standard pipeline separately, which produced more class-relevant gene sets during feature selection. The resulting putative cluster lists and visualizations aided manual inspection to (1) exclude remaining suspected doublet clusters and (2) merge clusters with extremely high similarity to neighboring clusters. This step resulted in the final cluster list. We then assigned a two-gene identifier name to each neuronal cluster, based on highly enriched genes and a literature survey.

##### Step 3: final feature selection and visualization

After Step 2 doublet removal and merging of similar clusters, we repeated only feature selection and replotted all visualizations, as presented in the study.

#### Standard clustering pipeline scRNA-seq

In each of the steps outlined above, we applied the following:

##### Normalization

Each cell (vector) was normalized to a length of 1, and then multiplied by 20,000.

##### Gene exclusion

We excluded 53 IEGs (*Btg2*, *Jun*, *Egr4*, *Fosb*, *Junb*, *Gadd45g*, *Fos*, *Arc*, *Nr4a1*, *Npas4*, *Coq10b*, *Tns1*, *Per2*, *Ptgs2*, *Rnd3*, *Tnfaip6*, *Srxn1*, *Tiparp*, *Ccnl1*, *Mcl1*, *Dnajb5*, *Nr4a3*, *Fosl2*, *Nptx2*, *Rasl11a*, *Mest*, *Sertad1*, *Egr2*, *Midn*, *Gadd45b*, *Dusp6*, *Irs2*, *Plat*, *Ier2*, *Rrad*, *Tpbg*, *Csrnp1*, *Peli1*, *Per1*, *Kdm6b*, *Inhba*, *Plk2*, *Ifrd1*, *Baz1a*, *Trib1*, *Pim3*, *Lrrk2*, *Dusp1*, *Cdkn1a*, *Pim1*, *Sik1*, *Frat2* and *Dusp*5), sex genes (*Xist*, *Tsix*, *Eif2s3y*, *Ddx3y*, *Uty* and *Kdm5d*) and genes that were not relevant (for example, when clustering neurons, all non-neuronal markers mentioned above were excluded).

##### Feature selection

First, we selected only genes with expression in at least five cells, but less than 50% of all cells. We then used CV versus mean fit to rank genes as described previously^74^. The number of genes selected was decided based on plotting the distance to the fitted line, from the largest to smallest distance, and finding the ‘bending’ point closest to the origin.

##### PCA

Next, we calculated PCA projections, deciding the number of PCs based on the optimal point of explained variance versus PC.

##### Batch correction

Because data collection was performed in three time-separated batches (Supplementary Table 1), we corrected for batch effects by applying the HARMONY algorithm^75^ to the PCA projection.

##### Dimensionality reduction by t-distributed stochastic neighbor embedding (t-SNE)

Two-dimensional embedding was performed using t-SNE (MATLAB implementation) with correlation distance, Barnes–Hut algorithm, *θ* = 0.5, learning rate = number of cells/12 and exaggeration = 20. The number of PCs was as determined above. To choose perplexity, we used the following heuristics: on PCA projections, we calculated the distance (correlation) of each cell to its first 500 neighbors and determined the optimal ‘cutting’ point. This produced an ‘optimal’ number of neighbors per cell, and we chose the perplexity to be the median of this vector. The t-SNE was initiated based on the first two dimensions of the PCA, as described in ref. ^76^. For t-SNE visualization of the final clusters (Step 3, as shown in the figures of the article), we used perplexity = 100 and exaggeration = 5.

##### Clustering

Next, we used the DBSCAN algorithm^77^ to cluster the t-SNE-embedded cells. We chose DBSCAN parameters based on visual inspection of the resulting clusters, aiming to avoid over-clustering, without losing the sensitivity to detect small clusters.

##### Postclustering

After clustering, we removed cells that DBSCAN labeled as outliers and sorted the cells in each cluster using one-dimensional (1D) t-SNE (applied to each cluster separately).

##### Dendrogram construction

To build the cluster dendrograms (a 1D order of clusters), first, we log_2_(*x* + 1)-transformed the expression; second, calculated cluster-wise mean expression profiles; third, calculated the PCA projection of the matrix; fourth, used the projection coordinates for linkage clustering (ward algorithm, correlation distance); and finally, used the MATLAB function ‘optimalleaforder’ to order the clusters.

##### Branch point marker gene identification

To help us (1) identify genes enriched in multiple clusters and (2) define main splits along the cell-type hierarchy, we computed branch point marker genes. At each junction (or split) along each class dendrogram, we calculated the difference of fraction positive cells on either branch of the junction. The resulting top-scoring genes, on either side, were considered branch point markers.

### ST Visium (10x Genomics)

For STs, two adult mice (one male and one female) were sacrificed by transcardial perfusion with aCSF. We quickly extracted, coated and cryomold-embedded the fresh brains in cryoprotective OCT (TissueTek), flash-froze them in isopentane equilibrated on dry ice and maintained them in sealed bags at −80 °C until processing. Per brain, we collected four right-hemisphere coronal cryosections at 10-μm thickness, aiming at approximately 200–300 μm spacing, spanning the anterior–posterior axis of the amygdala, onto the Visium ST gene expression slide (four capture areas, 10x Genomics). For tissue preparation, we followed the manufacturer’s instructions, with the following specifications: methanol fixation, immunofluorescence staining with DAPI only (no antibody), imaging at ×4 on a Nikon Eclipse Ti2, DIC, DAPI and TRITC channels for fiducial and section alignment and 25 min permeabilization, as we had previously determined on amygdala test sections, using the Tissue Optimization kit. We then proceeded with the Visium Gene Expression Kit following the manufacturer’s instructions, with 15 PCR cycles for cDNA amplification. Sequencing was performed on Illumina NGS platforms to a depth of 150–200 M reads per sample (that is, capture area or section).

### Visium ST alignment and quality control

To map the anatomical annotation for each 2D capture spot, we aligned the DAPI images of Visium ST sections to the Allen Reference Atlas—Mouse Brain (atlas.brain-map.org), correcting for distortion caused by sectioning along the dorsoventral and mediolateral axes. Sections were on average 100 μm apart, with considerable variability (*z*-axis resolution); mRNA-capture spot diameters were 55 μm, centers 100 μm apart (*x*–*y* resolution). An average of 3,226 capture spots covered each coronal hemisphere, 5–10% of which mapped to the amygdala (mean 272 spots). Spots that aligned with the amygdala contained ~2,500 to 3,500 genes and ~30,000 UMIs. Of the eight sections sampled, two were too anterior and are not shown.

### Quantification and statistical analysis of Visium ST data

With the exception of the spatial barcodes replacing cell barcodes (spots instead of cells), the analysis was similar to the scRNA-seq analysis pipeline described above. Each spatial barcoded spot of 55-μm diameter was expected to capture multiple cells (including neurons, glia and vasculature), resulting in ‘micro-bulk’ expression data. We previously found non-neuronal cells to be less spatially distinct in the brain^14^ and, therefore, expected genes derived from non-neurons to be noisy, sporadic and spatially less informative. To focus on neuronal diversity instead, we removed non-neuronal marker genes from the analysis.

Briefly, after preprocessing, filtering, normalizing and feature selection, we performed dimensionality reduction by principal component analysis (PCA) of the high-dimensional expression data, followed by 2D embedding with t-SNE, based on their similarity in the high-dimensional gene expression space. This resulted in a 2D map. Focusing on neuronal markers, we clustered these spots according to their distance in 2D, using the density-based spatial clustering of applications with noise (DBSCAN) algorithm. Thus, spots with similar gene expression were grouped together in the same cluster. We also used *k*-nearest neighbors to regroup the outlier spots obtained from the DBSCAN algorithm into their closest neighbor cluster. Next, we remapped the spots to their original location on the tissue, based on the spatial barcode index, and inspected whether the clusters followed an unbiased spatial distribution in the tissue.

### Visium ST-based spatial correlation analysis

We used Visium ST expression data to infer the spatial distributions of scRNA-seq annotated cell-type mean cluster expression. First, we performed feature selection of highly variable genes for scRNA-seq and Visium ST datasets separately, then combined and intersected the two-gene lists. After normalizing both datasets, we conducted pairwise linear Pearson correlations between each scRNA-seq cluster and each Visium spatial spot (2D). This resulted in a matrix of *ρ* correlation values for each spot with each cell type, which we mapped back to the original *xy* position of the relevant section. For global pattern comparison, we normalized the *ρ* correlation values and presented all the section spots together in a heatmap form. Having a registered anatomical annotation for each 2D capture spot (see above, ST Visium), we also quantified spatial correlations for each scRNA-seq cluster per region, as presented in Extended Data Fig. 2.

Finally, to visualize all neuronal cell types simultaneously and enable the identification of spatial patterns and relationships between cell types, or groups of related cell types (branches on the dendrogram), we generated a weighted colormap for the sections. The weighted colormap is created based on the scores obtained from the *ρ* correlation values of each cell in the branch. The weighting considers the scores of all cell types in every spot, reflecting their relative importance in the overall analysis. This procedure was to visualize all cell types simultaneously, or GABA, or GLUT cell types, only.

### Allen Mouse Brain in situ hybridization Atlas-based spatial correlation analysis

To align the gene expression profiles detected for each scRNA-seq-derived cluster with its spatial context, we performed correlation to the Allen Mouse Brain in situ hybridization Atlas, using quantified expression values as 3D grids available through the Allen Mouse Brain API (http://help.brain-map.org/display/mousebrain/API)^78^, as we described before^14^. Briefly, we used the AMBA aggregated data, which provide an ‘energy score’ for each gene from the in situ experiment, per each voxel of 200 × 200 × 200 μm. Each 200 μm voxel also has an AMBA-generated brain region annotation (the Atlas has a finer resolution of 25 μm voxels). Next, we calculated the *ρ* correlation of each cell type (average expression profile) and each voxel in the brain (left hemisphere) as follows: we defined genes that have a valid expression in the AMBA database as covering >30 voxels, a >5 ‘energy score’ and an average over all voxels >0.2. The set of quality genes was intersected with the set of genes from the final feature selection of our scRNA-seq data analysis (Step 3). The *ρ* correlation of each cell type with the region-annotated 200 μm voxels is presented in Supplementary Table 3. For better resolution and smooth visualization, we parcellated the coarse 200 μm voxels to a finer, linear interpolated 25 μm grid, and visualized resulting heatmaps on one coronal hemisphere.

### Multiplexed fluorescence in situ hybridization

Following the same procedure as for ST (10x Genomics Visium), we extracted aCSF-perfused brains, coated and cryomold-embedded them in cryoprotective OCT (TissueTek), flash-froze in isopentane equilibrated on dry ice and maintained in frozen brains in sealed bags at −80 °C until processing. We collected 16 μm coronal cryosections spanning the amygdala on Superfrost slides (Thermo Fisher Scientific), or 2% APTES silanized glass slides^79^, proceeded with quick postfixation in 4% PFA for 10 min, two washes in PBS, dehydration in isopropanol and stored slides in 70% ethanol at 4 °C until further processing. Before hybridization, sections were briefly dehydrated in 100% ethanol, airdried and encircled with a hydrophobic barrier pen. Starting with protease 4 pretreatment, we used the RNAScope Fluorescent Multiplex (3-plex) Reagent Kit (ACDBio) and followed the manufacturer’s instructions. The following mouse probes were combined for 3-plexing in alternating channels: Slc17a6 319171, Slc17a7 416631, Gad2 439371, Gal 400961, Avp, O2 472261, Sim1 526501, Krt9 454041, Fezf1 812321, Pvalb 421931, Pthlh 456521, Moxd1 497531, Grp 317861, Rspo2 402001, Cartpt 432001 and Penk 318761. Sections were imaged on a Nikon Eclipse Ti2 epifluorescence microscope at ×4 and ×20 magnification. Image processing was carried out using NIS Elements software (Nikon), and final images, LUTs 95th to 99th percentile, were batch exported using MATLAB. To map sections to anatomical annotations, we aligned DAPI images with the Allen Reference Atlas—Mouse Brain, adjusting for distortions along the dorsoventral and mediolateral axes caused by sectioning.

### Cell types spatial assignment

Based on the correlations with Visium ST, AMBA volumetric in situ hybridization expression data and multiplex in situ hybridization, most cell types could be mapped to amygdala subregions (or compartments), as described in the figures and text. We manually inspected neuronal cell types with low, or no, correlation within amygdala regions, and found the following two explanations: (1) accidental sampling of neighboring structures (for example, GABA-12, GABA-14 and GABA-15, CP; VGLUT1 16 and VGLUT1 17, ventral CA3 and DG; and GABA-25, globus pallidus) and (2) spatial correlation reached its detection limit, for example, GABA-25 had a unique gene signature of *Gpc5*, *Gbx1*, *Lhx8*, *Tacr3*, *Megf11*, *Th* and *Fibcd*, several of which located it to the neighboring pallidum (TELINH1 (ref. ^14^)). In the latter case (2), cell types were either restricted to a very small area (for example, Avp-Gal GABA-22, MEA and Oxt-Avp VGLUT2 type 32, SO; Fig. 3f), rare (for example, olfactory clusters VGLUT2 types 1–17 and Cajal Retzius cells VGLUT2 type 36) and/or dispersed (for example, canonical interneurons GABA-35 to GABA-56, which make up just 59% of neurons in mouse BLA^8^).

### Retrograde tracing and in situ hybridization

We used retro-AAVs ssAAV-retro/2-hEF1α-mCherry-WPRE-bGHp(A) (v212) and ssAAV-retro/2-hSyn1-chI-EGFP_2A_iCre-WPRE-SV40p(A) (146, VVF Zurich). The plasmids p146 and p212 were constructed by the VVF (iCre; Addgene, 24593). Mice were anesthetized and maintained under anesthesia using isoflurane (0.2%; SomnoSuite, Kent Scientific Corporation). The animals were placed on the stereotactic rig (Neurostar, Kopf Instruments), their body temperature was maintained at 37 °C and ophthalmic ointment (Duratears, Alcon Couvreur NV) was applied, the head was shaved, the scalp was disinfected, the skull was exposed and bregma–lambda was recorded to correct coordinates. retroAAV-tracer viruses were unilaterally injected into the MOB (AP, 4.5 mm; ML, 0.5 mm and DV, 1.8 mm) and BLA (AP, −1.46 mm; ML, 3.10 mm and DV, 4.95 mm), using pulled glass micropipettes (BRAND, disposable BLAUBRAND micropipettes, intra-Mark, 5 ml). Mice recovered from anesthesia in a heated chamber, and returned to group housing in HCs, with postsurgery monitoring and administration of painkiller buprenorphine (0.05–0.1 mg kg^−1^) for 2 d post surgery. For 2–3 weeks post surgery, mice were killed with an overdose of ketamine/xylazine, followed by transcardial perfusion with PBS, and 4% PFA. Brains were extracted, postfixed in 4% PFA overnight, cryoprotected in 30% sucrose, cryoembedded in OCT and stored at −80 °C until processing. We collected 50 μm cryosections, counterstained (NucBlue; Invitrogen, R37606), coverslipped (ProLong Diamond; Thermo Fisher Scientific, P36961) and imaged on a Nikon Eclipse Ti2 epifluorescence microscope (×4). Imaged sections were immediately stored at −80 °C. For in situ hybridization, slides were thawed, coverslips gently removed in PBS and processed as described above; starting with stepwise ethanol dehydration (50% ethanol followed by 70% ethanol, and finally 100% ethanol for 5 min each), and pretreatment in Protease 3 (RNAScope Fluorescent Multiplex (3-plex) Reagent Kit, ACDBio).

### Cell activity score by high IEG expression (IEG score)

We defined a set of the following eight IEGs: *Arc*, *Bdnf*, *Btg2*, *Fos*, *Fosl2*, *Homer1*, *Npas4* and *Nr4a1*, and calculated the 90th percentile expression for each. A cell was called activated, or IEG^high^, if it expressed any of the genes above this 90th percentile. For every cell type, we calculated the fraction of activated cells per timepoint (8 × 5 matrix). We then summed the fraction of all eight genes per timepoint (1 × 5 vector) and defined the activity score as the maximum difference between CFC samples (2 h, 8 h, 24 h, recall) to HC control.

### Differential gene expression of activated neurons (IEG^high^)

Based on the same set of eight IEGs (*Arc*, *Bdnf*, *Btg2*, *Fos*, *Fosl2*, *Homer1*, *Npas4* and *Nr4a1*), we consider a cell ‘activated’ or IEG^high^, if its expression is in the top 5% of the gene expression distribution for any of these IEGs (95th percentile over all cells). For all CFC-sampled cells per cell type, we then performed differential gene expression analysis for IEG^high^ versus IEG^low^ cells. We calculated the average expression of each group and plotted the difference versus average (Fig. 6g). For each cell type, we registered the value of the difference ($d$IEG^high^–IEG^low^) = and ranked genes by their cluster frequency of differential expression ($d > 0.5$) in their respective class (GABA, GLUT). Gene Ontology (GO) terms enriched IEG^high^ for IEG^low^ cells per class were analyzed in DAVID biological processes BP5 (Fig. 5 and Extended Data Fig. 8).

### Analysis of CFC-associated gene expression correlation (correlation score)

We defined the following seven groups of genes indicated in the literature to be related to neuronal activity-dependent transcription (Supplementary Table 4): IEG, learning/memory, glutamate receptors, GABA receptors, K^+^-channels, Ca^2+^-channels and semaphorins. We included genes expressed in more than 200 cells per analyzed class, and cell types with $\ge$20 cells per sampling timepoint. Per cell type, per timepoint, we then calculated Pearson pairwise correlation for each gene list, and all gene lists combined (correlation summary). Based on correlation value and sample size, we transferred correlation values to two-sided *P* values. From this, the correlation score for each cell type and timepoint was calculated (${\rm{score}}=-{\log }_{10}(p)$). The per-cluster correlation score (Fig. 6d) was the difference between the maximum score among CFC (2 h, 8 h, 24 h or recall) and the maximum score of HC control (batch A or B). For *Nlgn1* and *Bdnf* (GLUT only), we calculated Pearson pairwise correlations (per timepoint, per cluster), with all genes expressed in >5 cells, and ranked correlated genes (*P* < 0.01, two-sided) by their frequency of correlation in CFC. GO terms enriched among the top 200 correlated genes were analyzed in DAVID biological processes BP5 (Extended Data Fig. 9).

### In vivo fiber photometry recordings

#### Stereotactic surgery for viral injection and optic-fiber implantation

Mice were anesthetized with intraperitoneal injection of a cocktail of ketamine (0.1 mg g^−1^) and dormitor (0.01 mg g^−1^) and maintained under anesthesia using isoflurane (0.2%; SomnoSuite, Kent Scientific Corporation). The animal was placed on the stereotactic rig (Kopf Instruments), their body temperature was maintained at 37 °C, and ophthalmic ointment (Duratears, Alcon Couvreur NV) was applied. The head was shaved, the scalp was disinfected, and the skull was exposed and leveled using bregma–lambda measurements. The region of interest was marked and holes were drilled for viral injection, optic-fiber implantation and fixing of supporting screws.

We used retro-ssAAV-retro/2-hSyn1-chI-GCaMP6f-WPRE-SV40p(A) (V83) (VVF Zurich). The plasmid p83 was constructed by the VVF (GCaMP6f: Addgene, 51085). retroAAV-GCaMP6f virus was unilaterally manually injected into the MOB (AP, 3.2 mm; ML, 0.5 mm and DV, 3 mm), using a pulled glass micropipette (BRAND, disposable BLAUBRAND micropipettes, intra-Mark, 5 ml).

Following the viral infusion in MOB, optic fiber (Doric lenses, 200 µm, NA 0.66, 6 mm long, zirconia ferrule, flat-fiber tip) implantation was performed in the ipsilateral NLOT (AP, −0.46 mm; ML, 2 mm and DV, 5.2 mm) to record calcium signals. Screws and optic fiber were fixed to the skull plate using dental cement (UNIFAST, GCAmerica). Mice recovered from anesthesia after injection of Antisedan (subcutaneous (s.c.) 0.1 ml 10 g^−1^), with postsurgery monitoring and administration of painkiller Norocarp (0.005 mg g^−1^) and antibiotic Baytril (0.03 ml 10 g^−1^) for 3 d. Behavioral testing and recordings were done 3–4 weeks post surgery.

#### Fiber photometry recording and synchronization

Calcium signals were recorded using the RZ10x system of Tucker Davis Technologies and an optical path by Doric, which includes 600 µm mono-fiber optic patch cords connected to a four ports Fluorescence MiniCube (FMC4_IE(400-410)_E(460-490)_F(500-550)_S) and a 200 µm optical patch cord with a fiber-optic rotary joint (FRJ_1×1_PT_200/230/LWMJ-0.57_1m_FCM_0.15m_FCM) connected to the recorded animal.

A high-quality monochromatic camera (Flea3 USB3, FLIR), equipped with a wide-angle lens, was placed at the top of the acoustic chamber and connected to a computer, enabling a clear view and recording (∼30 frames per second) of the subject’s behavior using commercial software (FlyCapture2, FLIR). The camera (Flea3 USB3) was connected to the digital port of the RZ10× system and configured to deliver strobes for every frame acquired. These were later used to synchronize the video frames with the calcium signal. TDT Synapse software (TDT) was used for recording the GCamp signal channel (excitation 470 nm), the isobestic control channel (405 nm) and the digital channel receiving the camera strobes.

All fiber photometry recordings were performed in the CFC apparatus described above. The optic fiber of the implanted mouse was connected to the optical patch cords via a sleeve connector (Doric) under mild isoflurane anesthesia. The animal was then allowed to habituate in the behavior setup for 15 min. Recording took place during the last 5 min of habituation (baseline), which was followed by tone-shock pairing (7 min) or a recall session (5–6 min) conducted 24 h later.

#### Data analysis

Animals that did not show viral expression or proper placement of optic fiber in histology were excluded from the subsequent data analysis (three of eight mice). Calcium signal data was analyzed using a custom-written MATLAB Script. First, we fitted the 405 channels onto 465 channels to detrend signal bleaching and any movement artifacts, according to the manufacturer’s protocol (https://github.com/tjd2002/tjd-shared-code/blob/master/matlab/photometry/FP_normalize.m). Next, the signal was aligned to the video recording using the timestamps recorded by the digital port of the RZ10× system. We aligned the calcium signal to each event and normalized it using *z* score (0.5 s bins), where the 5-s pre-event period served as the baseline.

### Statistics and reproducibility

Individuals and experimental groups for scRNA-seq are reported in Supplementary Table 1. No sample size calculation was performed. We included two to four biological replicates for each sampling condition after cued fear conditioning. Mice were randomly assigned to naïve, 2 h, 8 h, 24 h or recall. Blinding was not possible for CFC, but data were analyzed blinded, using automated scripts. Neurons below 3,000 UMI per cell, or 2,500 genes per cell, and doublets were excluded from the analysis. Data distribution was not assumed to be normal, and no statistical tests assumed normality. Histological validation of cell types and gene expression (multiplex in situ hybridization) was carried out in a minimum of two relevant anterior–posterior sections, from a minimum of two individuals, each. For retrograde-AAV projection tracing of the NLOT, we first calibrated virus labeling and stereotaxic coordinates in three mice, then replicated projection labeling in four individuals. Images show consecutive sections from one representative mouse. In fiber photometry, we excluded three of eight mice that underwent surgery and recordings, due to no verified expression of GCaMP and/or incorrect fiber optic placement.

### Reporting summary

Further information on research design is available in the Nature Portfolio Reporting Summary linked to this article.

## Online content

Any methods, additional references, Nature Portfolio reporting summaries, source data, extended data, supplementary information, acknowledgements, peer review information; details of author contributions and competing interests; and statements of data and code availability are available at 10.1038/s41593-023-01469-3.

### Supplementary information

Reporting Summary

Supplementary Tables Supplementary Table 1: scRNA-seq samples and experimental metadata. Supplementary Table 2: Cluster-wise gene expression enrichment for neuronal types. Supplementary Table 3: Region-wise correlation of neuronal types to AMBA volumetric gene expression (http://help.brain-map.org/display/mousebrain/API). Supplementary Table 4: List of genes used for analysis of learning-related gene modules. Supplementary Table 5: Differentially expressed genes between 2 h and recall timepoints, per neuron class (GABA and GLUT), and associated GO terms using DAVID biological processes 5.

### Source data

Source Data Fig. 1 Lists and fraction enrichment of genes differentially expressed along the dendrogram’s hierarchical splits; for every branch (‘left and right’), on every junction of the dendrogram. A subset of genes is displayed as a heatmap in Fig. 1c (Methods—Branch point marker gene identification).

Source Data Fig. 4 List of neuronal cell types, with IEG score (Methods), cluster size (cells) and smallest group size (cells).

Source Data Fig. 5 List of top genes upregulated in IEG^high^ fractions, per GABA and GLUT class, with GO terms using DAVID biological processes 5.

Source Data Fig. 6 Correlation scores (Methods) per gene module indicated, for each cell type, in GABA and GLUT. Cell types with small group sizes are marked 0.

Source Data Extended Data Fig. 2 Region-wise correlation of neuronal types to Visium spatial transcriptomics, registered to the Allen Reference Atlas—Mouse Brain (atlas.brain-map.org).

Source Data Extended Data Fig. 8 List of top genes downregulated in IEG^high^ fractions, per GABA and GLUT class, with GO terms using DAVID biological processes 5. Source data for upregulated genes is included in Fig. 5.

Source Data Extended Data Fig. 9 Genes correlated with the expression of Bdnf, or Nlgn1, per neuron class indicated, with GO terms using DAVID biological processes 5.

## Data Availability

The sequencing data generated in the current study is available in the ArrayExpress database at EMBL-EBI, under accession E-MTAB-12096. For convenience, the final single-cell expression dataset with annotations and metadata is available as a table at figshare: 10.6084/m9.figshare.20412573. Source data files for figures are available alongside the manuscript where appropriate. We used the Allen Reference Atlas—Mouse Brain to align and annotate brain regions, available at http://atlas.brain-map.org/. We used the Allen Mouse Brain ISH Atlas^78^ (available from https://mouse.brain-map.org) for in situ hybridization images of several individual genes. For spatial cell-type correlation, we used the quantified expression values as 3D grids available through the Allen Mouse Brain API (http://help.brain-map.org/display/mousebrain/API). We provide an online browsable resource of single-cell expression data, and spatial distributions of cell types, available at https://zeisellab.org/amygdala/. Source data are provided with this paper.

## Code availability

Custom code used to perform the analysis is available at https://github.com/zeiselamit/amygdala_fc.
