# Author Correction: Neuronal types in the mouse amygdala and their transcriptional response to fear conditioning

**DOI:** 10.1038/s41593-024-01578-7

**Published:** 2024-01-19

**Authors:** Hannah Hochgerner, Shelly Singh, Muhammad Tibi, Zhige Lin, Niv Skarbianskis, Inbal Admati, Osnat Ophir, Nuphar Reinhardt, Shai Netser, Shlomo Wagner, Amit Zeisel

**Affiliations:** 1 Faculty of Biotechnology and Food Engineering, Technion—Israel Institute of Technology, Haifa, Israel; 2 Sagol Department of Neurobiology, University of Haifa, Haifa, Israel

**Keywords:** Fear conditioning, Molecular neuroscience

Correction to: *Nature Neuroscience* 10.1038/s41593-023-01469-3, published online 26 October 2023.

In the version of the article initially published, the sentence “Scale bars, overview, 1 mm; zoom-ins, 100 mm” was missing from the Fig. 3 legend. Additionally, the units on three of the scale bars in Fig. 6e and Extended Data Fig. 6c and d have been corrected to “µm”. The errors have been corrected in the HTML and PDF versions of the article.

