# Supplementary material for: Neuronal types in the mouse amygdala and their transcriptional response to fear conditioning
Source: Nat Neurosci. 2023 Oct 26;26(12):2237–49. doi: 10.1038/s41593-023-01469-3 (PMC10689239; doi:10.1038/s41593-023-01469-3)
Supplement: Supplementary file 1 — Reporting Summary [file 41593_2023_1469_MOESM1_ESM.pdf]

nature portfolio

Corresponding author(s): Amit Zeisel

Last updated by author(s): Sep 6, 2023

Reporting Summary

Nature Portfolio wishes to improve the reproducibility of the work that we publish. This form provides structure for consistency and transparency in reporting. For further information on Nature Portfolio policies, see our Editorial Policies and the Editorial Policy Checklist.

Statistics

For all statistical analyses, confirm that the following items are present in the figure legend, table legend, main text, or Methods section.

n/a	Confirmed	
☐	☒	The exact sample size (*n*) for each experimental group/condition, given as a discrete number and unit of measurement
☐	☒	A statement on whether measurements were taken from distinct samples or whether the same sample was measured repeatedly
☐	☒	The statistical test(s) used AND whether they are one- or two-sided<br>*Only common tests should be described solely by name; describe more complex techniques in the Methods section.*
☒	☐	A description of all covariates tested
☐	☒	A description of any assumptions or corrections, such as tests of normality and adjustment for multiple comparisons
☐	☒	A full description of the statistical parameters including central tendency (e.g. means) or other basic estimates (e.g. regression coefficient) AND variation (e.g. standard deviation) or associated estimates of uncertainty (e.g. confidence intervals)
☐	☒	For null hypothesis testing, the test statistic (e.g. *F*, *t*, *r*) with confidence intervals, effect sizes, degrees of freedom and *P* value noted<br>*Give P values as exact values whenever suitable.*
☒	☐	For Bayesian analysis, information on the choice of priors and Markov chain Monte Carlo settings
☒	☐	For hierarchical and complex designs, identification of the appropriate level for tests and full reporting of outcomes
☒	☐	Estimates of effect sizes (e.g. Cohen's *d*, Pearson's *r*), indicating how they were calculated

Our web collection on statistics for biologists contains articles on many of the points above.

Software and code

Policy information about availability of computer code

Data collection	Fiber photometry; We used FlyCapture2 (FLIR) to track mouse behavior, and TDT Synapse (TDT Inc.) to record both the GCamp signal channel (excitation 470 nm), the isobestic control channel (405 nm) and the digital channel receiving the camera strobes.
Data analysis	CellRanger 5.0.1, Matlab 2022a (including, e.g., tSNE (Barnes Hut), Wards), DBSCAN (Ester et al. 1996), HARMONY (Korsunsky et al. 2019), Gene Ontology (DAVID, biological processes (BP5)), NIS Elements software (5.10.01). Custom code is available at https://github.com/zeiselamit/amygdala_fc<br>Fiber photometry: According to the manufacturer's instructions, we de-trended, and corrected movement artefacts based on isobestic control, using https://github.com/tjd2002/tjd-shared-code/blob/master/matlab/photometry/FP_normalize.m

For manuscripts utilizing custom algorithms or software that are central to the research but not yet described in published literature, software must be made available to editors and reviewers. We strongly encourage code deposition in a community repository (e.g. GitHub). See the Nature Portfolio guidelines for submitting code & software for further information.

Data

Policy information about availability of data

All manuscripts must include a data availability statement. This statement should provide the following information, where applicable:
- Accession codes, unique identifiers, or web links for publicly available datasets
- A description of any restrictions on data availability
- For clinical datasets or third party data, please ensure that the statement adheres to our policy

The sequencing data generated in the current study is available in the ArrayExpress database at EMBL-EBI, under accession E-MTAB-12096. For convenience, the final single-cell expression dataset with annotations and metadata is available as a table at figshare: https://doi.org/10.6084/m9.figshare.20412573 . Source Data files for Figures are available alongside the manuscript where appropriate. We provide an online browsable resource of single-cell expression data, and spatial distributions of cell types, available at https://zeisellab.org/amygdala/. We used the Allen Reference Atlas - Mouse Brain to align/annotate brain regions. It is available at http://atlas.brain-map.org/. We used the Allen Mouse Brain Atlas published in Lein et al. 2014 (available from mouse.brain-map.org) for in situ hybridization images of several individual genes, and for spatial cell type correlation, the quantified expression values as 3D grids available through the Allen Mouse Brain API (http://help.brain-map.org/display/mousebrain/API).

Human research participants

Policy information about studies involving human research participants and Sex and Gender in Research.

Reporting on sex and gender	N/A
Population characteristics	N/A
Recruitment	N/A
Ethics oversight	N/A

Note that full information on the approval of the study protocol must also be provided in the manuscript.

Field-specific reporting

Please select the one below that is the best fit for your research. If you are not sure, read the appropriate sections before making your selection.

☒ Life sciences ☐ Behavioural & social sciences ☐ Ecological, evolutionary & environmental sciences

For a reference copy of the document with all sections, see nature.com/documents/nr-reporting-summary-flat.pdf

Life sciences study design

All studies must disclose on these points even when the disclosure is negative.

Sample size	scRNA-seq: No sample size calculations were performed. We included 2-4 biological replicates for each sampling condition after cued fear conditioning (CFC) and targeted a sampling depth of total >30,000 neurons, similar to sampling sizes reported in previous publications, but limited by the significant costs for scRNA-seq. We report on CFC-results for individual clusters only when the number of cells per group were sufficient, and mark clusters where group size was sufficient, but small, throughout the figures and manuscript.
Data exclusions	scRNA-seq: We included high-quality cells only; defined by >3000 UMI/cell, >2500 genes/cell and excluded doublets, see Methods. For CFC-analysis, we included samples from 2 coherent experimental batches, with sufficient sampling depth, see Suppl. Table 1.<br>Fiber photometry: We excluded 3 of 8 animals that underwent surgery and recordings due to no verified expression of GCaMP and/or incorrect fiber optic placement, verified in histology.
Replication	Findings from scRNA-seq were replicated in individual samples. All data was pooled and analyzed together. We validate cell types with independent methods (ISH, multiplex FISH, Visium ST).<br>Histological validation of cell types and gene expression (multiplex in situ hybridization) was carried out in a minimum of 2 relevant anterior-posterior sections, from a minimum of 2 individuals, each.<br>For retrograde-AAV projection tracing of the NLOT, we first calibrated virus labelling and stereotaxic coordinates in 3 mice, and then replicated projection labeling in 4 individuals.<br>Fiber photometry was performed on 8 individuals. The results of 5 mice with verified GCaMP expression and correct fiber optic placement are reported.
Randomization	Mice were randomly assigned to naive, 2h, 8h, 24h or recall cohorts. No other randomization was carried out.
Blinding	Full blinding was not logistically possible during CFC, sample collection and dissection. scRNA-seq, alignment and cell-typing were carried out in a blinded fashion. Automated scripts were used for all analysis.

Reporting for specific materials, systems and methods

We require information from authors about some types of materials, experimental systems and methods used in many studies. Here, indicate whether each material, system or method listed is relevant to your study. If you are not sure if a list item applies to your research, read the appropriate section before selecting a response.

Materials & experimental systems

n/a	Involved in the study
☒	☐ Antibodies
☒	☐ Eukaryotic cell lines
☒	☐ Palaeontology and archaeology
☐	☒ Animals and other organisms
☒	☐ Clinical data
☒	☐ Dual use research of concern

Methods

n/a	Involved in the study
☒	☐ ChIP-seq
☒	☐ Flow cytometry
☒	☐ MRI-based neuroimaging

Animals and other research organisms

Policy information about studies involving animals; ARRIVE guidelines recommended for reporting animal research, and Sex and Gender in Research

Laboratory animals	Mus musculus, 7-12 week old males and females, C57Bl/6JOlaHsd or C57Bl/6J.
Wild animals	The study did not involve wild animals.
Reporting on sex	CFC and scRNA-seq: 22 males, 1 female. All mice are included in the cell type atlas. To minimize group sizes for fear conditioning, we used data from two experimental batches, from males only. Sample information is detailed in Suppl. Table 1.<br>Visium ST was performed on 1 male and 1 female.<br>All fiber photometry was performed on males.<br>Retrograde tracing was performed on females.
Field-collected samples	The study did not involve samples collected from the field.
Ethics oversight	All experimental procedures followed the legislation under the Israel Ministry of Health - Animal Experiments Council and were approved by the institutional Animal Experiments Ethics Committees at the Technion Israel Institute of Technology and Haifa University

Note that full information on the approval of the study protocol must also be provided in the manuscript.
